# Recent advances in two-dimensional perovskite materials for light-emitting diodes

**DOI:** 10.1186/s11671-024-04044-2

**Published:** 2024-07-02

**Authors:** Deepika Tyagi, Vijay Laxmi, Nilanjan Basu, Leelakrishna Reddy, Yibin Tian, Zhengbiao Ouyang, Pramoda K. Nayak

**Affiliations:** 1 Key Laboratory of Optoelectronics Devices and Systems of Ministry of Education and Guangdong Province, College of Electronic Science and Technology of Shenzhen University, THz Technical Research Center of Shenzhen University, Shenzhen University, Shenzhen, 518060 China; 2 Department of Physics, Indian Institute of Technology Madras, Chennai, 600036 India; 3 Department of Physics, University of Johannesburg, Johannesburg, 2006 South Africa; 4 2D Materials Research and Innovation Group, Indian Institute of Technology Madras, Chennai, 600036 India; 5 Centre for Nano and Material Sciences, Jain (Deemed-to-be University), Jain Global Campus, Kanakapura, , Bangalore, Karnataka 562112 India; 6 College of Mechatronics and Control Engineering, Shenzhen University, Shenzhen, 518060 China

**Keywords:** LED, Low-dimensional perovskite, Two-dimensional perovskites, Semiconductor materials, Graphene, Quantum dots, Hexagonal boron nitride

## Abstract

Light-emitting diodes (LEDs) are an indispensable part of our daily life. After being studied for a few decades, this field still has some room for improvement. In this regard, perovskite materials may take the leading role. In recent years, LEDs have become a most explored topic, owing to their various applications in photodetectors, solar cells, lasers, and so on. Noticeably, they exhibit significant characteristics in developing LEDs. The luminous efficiency of LEDs can be significantly enhanced by the combination of a poor illumination LED with low-dimensional perovskite. In 2014, the first perovskite-based LED was illuminated at room temperature. Furthermore, two-dimensional (2D) perovskites have enriched this field because of their optical and electronic properties and comparatively high stability in ambient conditions. Recent and relevant advancements in LEDs using low-dimensional perovskites including zero-dimensional to three-dimensional materials is reported. The major focus of this article is based on the 2D perovskites and their heterostructures (i.e., a combination of 2D perovskites with transition metal dichalcogenides, graphene, and hexagonal boron nitride). In comparison to 2D perovskites, heterostructures exhibit more potential for application in LEDs. State-of-the-art perovskite-based LEDs, current challenges, and prospects are also discussed.

**Graphical Abstract:**

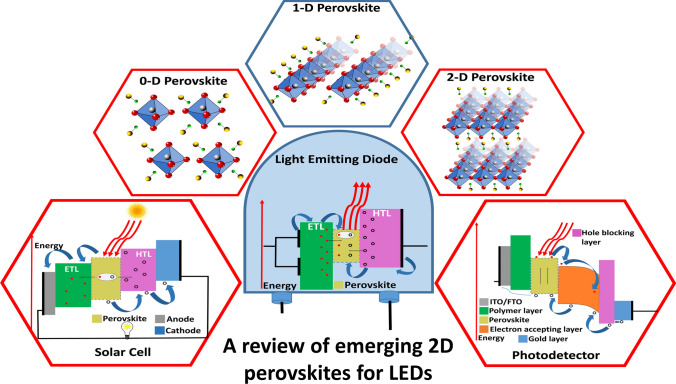

## Introduction

Devices that can radiate light on the application of an electric field have been referred to as electroluminescent devices. Light emitting diode (LED) comprises the bulk of electroluminescent devices and has been considered the preferred device that is more efficient than the conventional incandescent bulb or fluorescent lamps. This research topic has been advanced by the increased understanding of quantum mechanics, solid-state physics, and the implementation of alternative materials in manufacturing. LED devices are composed of various layers, the most important being the emissive layer (EML), which emits light by passing an electric current through it. Metal halide perovskites with the chemical formulae ABX_3_(A = Cs, B = Pb, X = I) have been incorporated in the implementation of displays in TVs and smartphones, and exhibit low performance when compared to organic LEDs. This issue leads to the development of low-dimensional perovskite-based LEDs (PeLEDs), which use various low-dimensional structural units linked on a plane or in a linear manner with the perovskite crystal. Hence, better light-emission performance is achieved which is structured around the quantum confinement effect of excitons [1, 2]. Furthermore, utilizing the surface-plasmon resonance properties of nanostructures and heterostructures of noble metal in the form of core shell or selectively doped nanoparticles significantly enhances the power density by acting as a carrier injector and provides a protective envelope to the LED devices [3, 4].

Materials forming the basis of LED systems are under constant investigation and advancement in terms of their direct or indirect applications for their sustainability in various environments. Thus, understanding material properties along with their current application and performance within optical devices is crucial for further advancement in LED systems. The conventional 2D materials forming the basis of LED systems are graphene, black phosphorous, transition-metal dichalcogenides (TMDs), and many more, which have already been explored and exploited for their compatibility in optical devices [5].

The application of graphene ranges from the photodiode to LASERs as graphene’s optical and electronic properties change with structural variations [6, 7]. Graphene is used in ultrafast pulsed LASER as it can generate extremely short pulses of light, whereas composite and stacked layers of graphene help in improving the stability, tuning capability, and efficiency of ultrafast LASER [8, 9]. In LASERs, the locking of modes and tuning of wavelength have been achieved by the incorporation of graphene layers via a doping mechanism [10, 11]. On the other hand, broadband and ultra-broadband range photodetectors have been designed by exploiting graphene’s excellent electrical conductivity, high carrier mobility, strong light-matter interaction, and low material [12, 13]. LASER applications with black phosphorous as a base material are used in the form of layered assembly [14, 15], exfoliated state [16, 17], or incorporated with polymers [14, 18]. Avenues of the photonic and sensing mechanism using black phosphorous are being continuously explored for better performance and stability [19, 20]. Other classes of materials used for their photonic applications range from MoS_2_ [21], h-BN, and organic–inorganic halide framework. A detailed study of the material properties and their characteristics is essential for choosing suitable materials for LED fabrication in enhancing their performances. In this review article, different sections focus on available semiconductor materials with working wavelength, advances in LED material technology, advances in fabrication technologies, and progress made in perovskite material-based LEDs. Several current works have been incorporated into this study which are related to 0-D, 1-D, and 2-D perovskite materials. Finally, we discuss the state-of-the-art perovskite-based LEDs (PeLEDs), and their current challenges, and prospects.

### Types of LED materials

Various solid-state lighting materials are gaining popularity due to many factors such as quality improvement, cost reduction, and lighting efficiency as mentioned in Table 1. The use of different types of phosphor materials in LEDs affects their chromaticity [22]. For instance, the degradation of phosphor plates resulting from irradiation of blue light increases their thermal properties thus consequently lowering the phosphor’s efficiency which changes the LED color rendering index [23]. The use of phosphor material in LED technology enables the absorption and emission of radiation energy in different portions of the electromagnetic spectrum. The use and control of multiple excitation sources and phosphor emissions facilitate specific light color provision including white light [24]. Various types of LEDs are discovered and acknowledged for their applications and properties.

**Table 1:** LEDs with a range of wavelengths, colors, threshold voltage, and the comprised materials

Wavelength range (nm)	Color	Threshold voltage (ΔV)	Semiconductor materials
< 400	Ultraviolet	3.1–4.4	AlN
			AlGaN
			AlGaInN
400–450	Violet	2.8–4.0	InGaN
450–500	Blue	2.5–3.7	InGaN, SiC
500–570	Green	1.9–4.0	GaP, AlGaInP, AlGaP
570–590	Yellow	2.1–2.2	GaAsP, AlGaInP, GaP
590–610	Orange	2.0–2.1	GaAsP, AlGaInP, GaP
610–760	Red	1.6–2.0	AlGaAs, GaAsP, AlGaInP, GaP
> 760	Infrared	> 1.9	GaAs, AlGaAs

*Conventional inorganic LEDs:* Since 1960, the traditional diode has been manufactured using different inorganic materials, i.e., gallium arsenide phosphide (GaAsP), aluminum gallium arsenide (AlGaAs), and others. The colors of the LED lights are generally based on the materials used. Various types of LEDs are used like bicolor and multicolor LEDs, (which contain several individual LEDs turned on by different voltages), Surface mount LEDs, Alphanumeric LED displays, Flashing LEDs, and others. The other inorganic LED, which is heavily used is the high brightness LEDs (HBLEDs). The higher light output generated by the HBLED controls the power at higher current levels. The HBLED shows longer life and greater efficiency level, especially when being switched on and off several times [25].

*Organic LEDs:* Organic LEDs are the basic light-emitting diodes where an organic basis material is used. Generally, a thin film of organic material is printed onto a glass-made substrate or fabricated as large sheets over selective substrates. The electrical charges acquired by the imprinted pixels are carried by the semiconductor circuit causing them to glow. The field of LED technology has been growing significantly, incorporating the organic basis with improvement in their efficiency for widespread usage [26].

### Advancement in the LED material technology

The progress in lighting technologies has come a long way from their inception, in terms of their power output and compactness in their size. The study of longevity, power output throughout their operation, and cost of fabrication are the driving factors for research and development in the various aspects of LEDs. The primary field of research and development is the underlying material responsible for the performance and efficiency of an LED unit. The material used in LED fabrication suffers from various drawbacks such as concentration quenching during non-radiative absorption, general reliability, photo saturation, and many more. Careful study of material properties and problem-specific selection of the basic material can resolve several issues and enhance LED performance. Mn^4+^-complex fluoride phosphors such as K_2_TiF_6_:Mn^4+^ (PFT) and K_2_SiF_6_:Mn^4+^ (PFS) have characteristics such as reasonable absorption at the blue LED wavelengths and narrow red emission while indicating that they also exhibit high efficiency which makes them suitable materials for LED lighting systems [27, 28]. However, while using these phosphor materials in LEDs, the activator concentration significantly tunes efficiency levels thus signifying the need to ensure critical control [29]. The optimal activator concentration is the point at which the brightness and quantum efficiency (QE) begin to decrease [23].

Diodes in LEDs consist of thin layers of semiconductor material [30, 31]. One sheet has excess electrons while the other one has a deficit. However, significant developments in LED technologies have been made, which promote the change of the traditional III-V compound semiconductors to modern wide-bandgap semiconductors such as Gallium Nitride (GaN) and Silicon Carbide (SiC) [32, 33]. Consequently, these adoptions have resulted in enhanced reliability and efficiency. GaN substrates play a vital role in obtaining ultrahigh-brightness in white LEDs [34]. These capabilities are facilitated by characteristics such as high current density, which enable driving the LEDs and consequently reducing their resistance [30]. The scope of improvement in the lighting technologies in terms of efficiency, durability, and reliability of LED lights is still a challenging aspect of the LED technologies [32, 35].

Solid semiconductor materials have facilitated new lighting technologies thus shifting from the traditional use of fluorescent tube lighting with toxic mercury to more modern developments such as LED lighting [36]. According to Khan et al*.* [46], newer solid semiconductor lighting such as LEDs has been developed having advantages over fluorescent tube lighting [37].

The reflectance spectrum of material influences the optical property changes in LED technology under different conditions. Microcellular PET reflective materials in LEDs have multiple impacts on color shift and lumen decay [22]. In LED devices, the color shift relies on various factors such as die silicone or silicon-phosphor interface, change in the phosphor emission peak, caused by heating, aging of lenses, reflectors, and other optical materials [22, 38]. The use of layers of PET reflective material enhances the harnessing property of LED strips and thus improves its efficiency [39]. The reflective materials enable the creation of different light colors based on the needs of the global population, and thus, supporting new developments and improvements.

Polymethyl methacrylate (PMMA) material in LEDs has become popular over recent years [40]. The excellent properties of PMMA such as optical transparency, surface hardness (scratch-free), durability against radiation, rigidity, and strength make it suitable for LED devices. Such development in LEDs based on PMMA has encouraged research on the luminous efficacy of LED, and consequently availing data regarding color shift and chromaticity stability [41]. With the obtained data, researchers can identify and initiate improvements to the current light systems, resulting in new developments [42].

The performance and reliability of LEDs significantly rely on the stacking of materials. The use of silicon materials in LED packaging facilitates long-time stability and highlight transmission [43]. The cost of extraction and production of materials used in the development of LEDs has increased over the years, which impacts the manufacturing processes and also causes environmental hazards. The use of silicon quantum dots (SiQDs) in red-emitting phosphors semiconductors is environment friendly and cost effective [44, 45]. The use of alkyl silanes (SiH_4_) and silicon oxide improves the photoluminescence quantum yield (PLQY) in phosphor semiconductors and the excitation power, thus enhancing lighting quality [46, 47]. Therefore, increased adoption and use of these silicon materials will continuously ensure the increased development of LEDs at favorable prices and relatively low impacts on the environment.

The LED packaging requires protection of the LED chip from external factors such as humidity, heat, and mechanical damage, which can alter the LED’s efficiency [48]. Organo-siloxane resins and epoxy are vital encapsulant materials used in LED devices for packaging technologies [49].

### Advances in the fabrication of the LED device

**Fig. 1:**
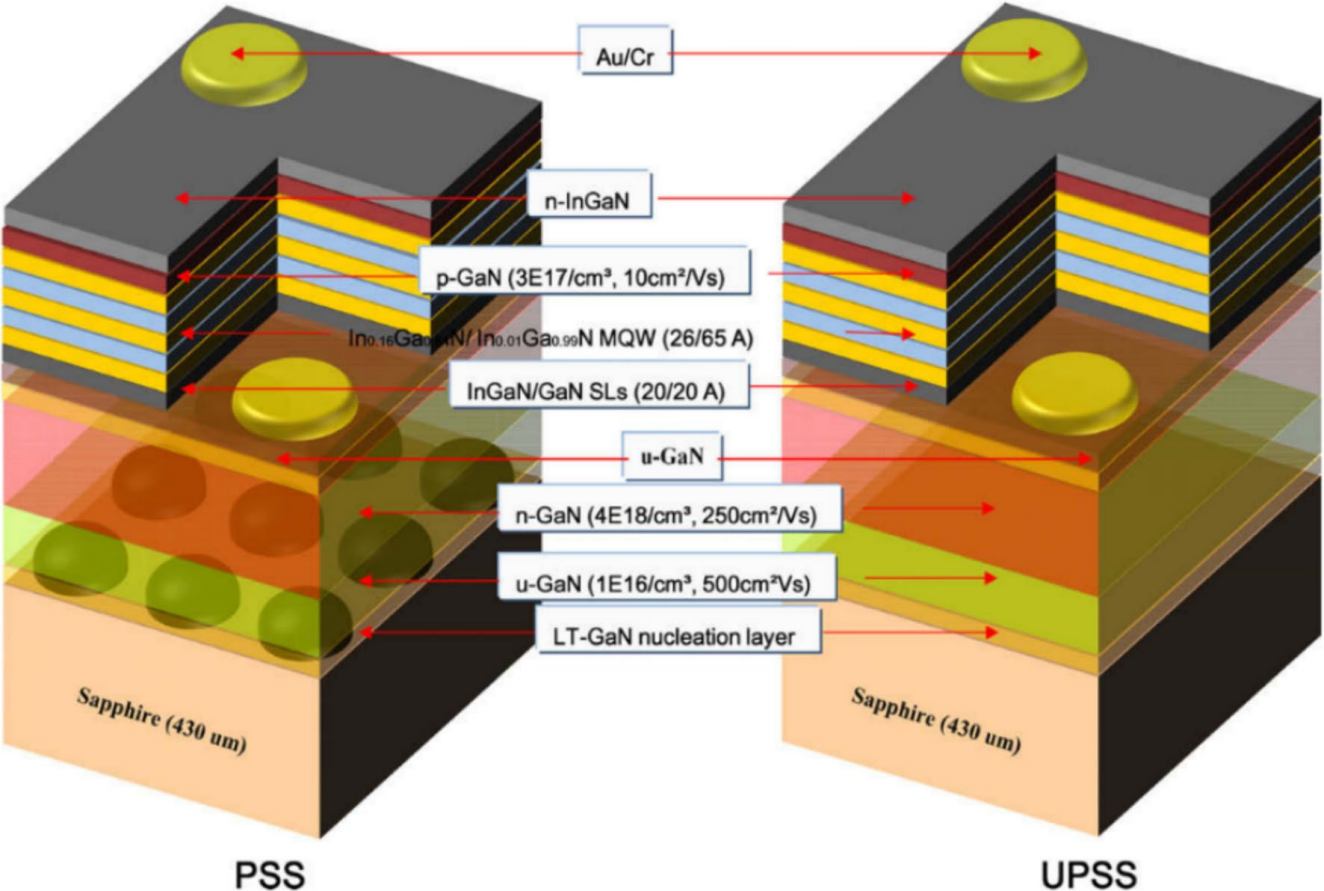
The schematic diagram of epitaxial layers and LED device structure using photonic crystals with material GaN Reproduced with permission from Ref. [50]. Copyright 2013, the IEEE Xplore publications

To improve LED device performance, multiple attempts have been made such as surface roughening of the indium thin oxide (ITO), using transparent electrode layer or p-Gan, using nano-tip arrays or ZnO nanorod, and using photonic crystals (PC). Despite the enhanced efficiency of these methods, the fabrication of nanostructures still occurs either directly or indirectly [50]. For instance, the fabrication of blue-light LEDs occurs through patterned sapphire substrates (PSSs). During this process, PSS acts as scattering centers of light in LED, while epitaxial lateral overgrowth enables the reduction of the epitaxial GaN layer’s threading dislocation density (Fig. 1). To design this kind of structure, photolithography/ electron beam lithography (EBL) techniques are used, conventional sapphire substrate (CSS) and dry-etched lens patterned sapphire substrate (LPSS) are also used [51].

Conventional microelectronic technologies are used in the fabrication of silicon nanostructure-based light emitting diodes (SiLED). SiLED are fabricated by injecting porous silicon and silicon-nanocrystals (Si-NCs) carriers to increase LED’s luminescent efficiency. The use of field effect electroluminescence mechanism involves the use of tunnel process in which hole and electron are alternatively injected into Si-NCs layer through a potential barrier. The ionization method involves the creation of carriers by hot electrons through the application of suitable voltage to the device’s electrodes, which facilitates the excitation of Si-NCs and thus improves the LED’s performance.

### Advances in perovskite material-based LEDs

**Fig. 2:**
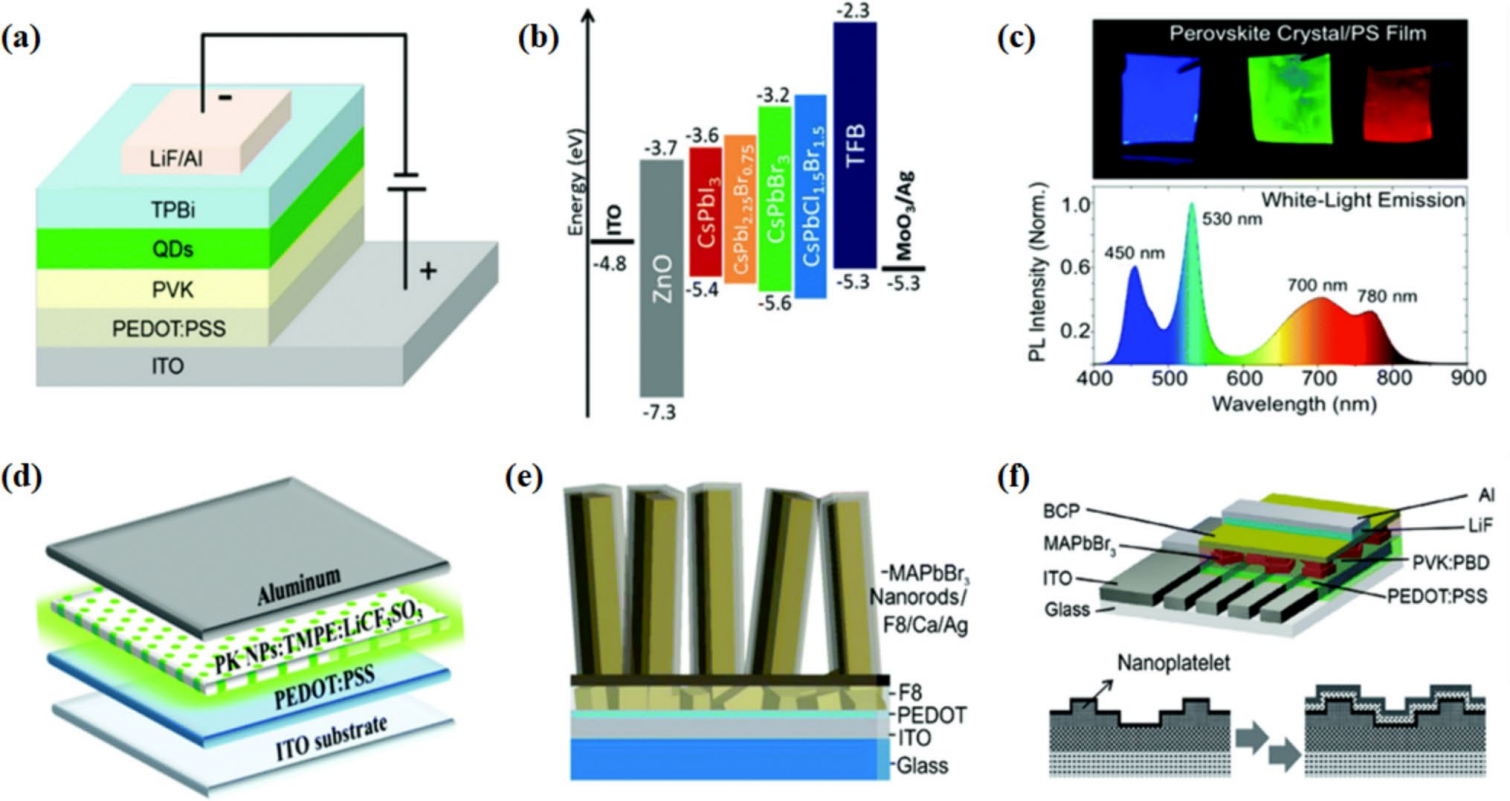
**a** A schematic diagram of a multilayer perovskite LED based on CsPbX_3_ Quantum Dots. **b** Energy level diagram for perovskite NC LEDs. **c** Perovskite crystals for emission of tunable white-light. **d** Light-emitting electrochemical cells based on perovskite nanoparticles prepared via spray coating. **e** Perovskite-nanorod-array LEDs are prepared via solution-phase growth and anion exchange conversion. **f** Perovskite nanoplatelet-based layer assisted by a bipolar polymer matrix reproduced with permission from Ref. [1]. © Copyright 2016, with the Royal Society of Chemistry publication

The use of conventional bulk perovskite materials in LED devices produces I-V hysteresis due to the migration of ionic defects present in the solution-processed thin films, leading to a lower yield in efficiency. To improve the device’s performance, grainless bulk structures (single crystals) or low-dimensional perovskite materials are used [52]. In an LED device, the emissive layer (EML) lies between the electron transport layer and hole transport layer and plays a primary role in making a good conducting mode of the device. The Perovskite QD LED standard device structure is shown below in Fig. 2. By comparing it with hybrid organic or inorganic perovskites, all inorganic perovskites offer enormous usage and higher stability in LED devices [53]. In LED and solar cell devices, the emissive layer (EML) is located in between the electron transport and hole transport layers and plays a primary role in making good conducting aspects of the device [54].

**Fig. 3:**
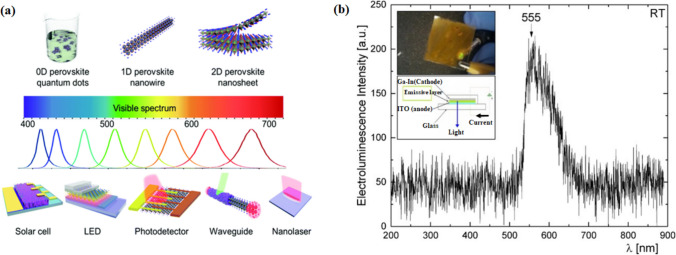
**a** Low dimensional perovskite’s applications based on atomic structure and wavelength reproduced with permission from Ref. [1]. **b** LED device electroluminescence spectra fabricated by (4-fluorophenethylamine-H)_2_PbI_4_ re-crystallized as thin film coating. Variation in light intensity has been seen. LED operating- Top image inset and Device schematic- bottom inset reproduced with permission from Ref. [57]. © Copyright 2016, with the Elsevier publication

Trimethyl aluminum (TMA) is used as a metal precursor in the atomic layer deposition technique, which shows the potential to improve the environmental stability in hybrid halide perovskite [55]. To fabricate the white light perovskite LEDs, the polymer matrix-assisted light emission strategy is used, as shown in Fig. 2, which shows a promising effort through perovskite nanostructure embedding with various emission colors in a suitable polymer material. Therefore, by tuning the traits of the electron layer and the hole transport layer in PeLEDs, there is a possibility of limiting the mentioned anomaly by making sure that the excitons can be confined to the emission layer. The entire device structure can thus be seen as a leveled-up low dimension material since the energy levels of the electron or hole levels are enough for exciton confinement. The 3-D PeLEDs have been seen to have a reduced operating voltage [56]. Furthermore, there have been developments into how the exciton-related properties of a material can be affected by the surrounding layers, and thus, it is important to understand how optical devices can be manipulated to provide better development. The high yield luminescent low-dimensional perovskite nanocrystal has been used in different applications like LEDs, nanolasers, waveguides, and photodetectors, as shown in the following Fig. 3a. For long-term stability and efficiency, there exist a lot of improvements available based on low-dimensional perovskites.

Recent developments have also shown that there is a growing interest in low-dimensional hybrid organic semiconductors for their application in LEDs that are functioning at normal room temperature [58]. This is achieved when the LED is structured around an ITO substrate of 4-flurophenethylamine-H_2_Pb layer. 2-D lead iodide perovskite proved to be simple and economical for LEDs, which works efficiently at 5─10 V and room temperature. The use of 3-D Hybrid Organic–Inorganic Semiconductor (HOIS) perovskite with the 2-D HOIS perovskite has shown beneficial properties like tunability of peak wavelength, good oscillator strength, and greater excitonic binding energy. HOIS are low-cost and self-assembled materials, which make them ideal for optoelectronic devices because of their inherent stable excitonic states at room temperature. The synthesis of (FpA-H)_2_PbI_4_ is reported to be synthesized for single layered LEDs (Fig. 3b)[57, 59]. The properties of 2-D hybrid organic–inorganic perovskites may be enhanced by the 2-D low dimensional semiconductors, which shall be useful in the complex hybrid LED production processes [35, 60, 61]. Particularly, the quasi-2-D analogues and 2-D HOIS can cover the spectra from the UV part with excitonic peaks, and thus make them comparable to 3-D HOIS. They tend to be more tunable, based on the added structure stoichiometry effect and other factors that affect the level of the excitonic peak [62, 63].

## Low dimensional perovskite material for leds

Low-dimensional nanostructures have become highly effective in the manufacturing of LEDs due to the physical and chemical aspects that allow them to be altered, thus having control over the dimensions between 1- 100 nm [64–66]. In nanostructures, definite dimensions can be achieved with new optical, magnetic, and electrical aspects that would not be seen in other voluminous materials. The atoms of the substance can exhibit varying properties by lowering the dimensions of the substance, the variation in surface-to-volume ratio can allow the substance to adopt new characteristics or features for applications in LED, photodetectors, biosensors, and solar cells [67–69]. A mesoporous ZnO/ZnAl2O4 mixed metal oxide (MMO) based Zn/Al layered double hydroxide (LDH) has been used as an effective anode material for visible light photodetectors. The device, assembled with Ruthenizer dye (535-bisTBA), shows significant photo-responsivity and specific photo-detectivity due to the high dye loading. The ZA-400 photodetector shows superior photo-responsive behavior compared to similar geometries, with an approximate response time of 900 ms [70]. Also, a new technique for preparing ZnO/ZnAl2O4-mixed metal oxide (MMO) as anode materials for visible light dye-sensitized photodetectors is presented. The study reveals that the high surface area of MMO anode materials enhances dye absorptivity, facilitating free electron transfer and increasing photocurrent. The optimized Z7A DS photodetector shows photo-responsivity and photo-detectivity [71]. A series of dye-sensitized solar cells (DSSCs) have been fabricated using mesoporous CuO@Zn(Al)O-mixed metal oxides and dye N719 as light absorbers. The DSSCs' power conversion efficiency is correlated with the dye loading amount, with CuO@MMO-550 showing a significant fill factor and power conversion efficiency of 0.55% and 1.24%, respectively [72]. A new technique for creating hexagonal-shaped mixed metal oxides (MMO) nanorods using Zn/Al-layered double hydroxide as a precursor is presented for dye-sensitized solar cell (DSSC) applications. The nanorods' diameter correlates with DSSC efficiency, and the optical behavior and absorption enhancement are demonstrated. The nanorods' diameter increases due to the enhanced surface area, allowing for higher dye loading, leading to improved short-circuit current. ZA-8 exhibits the highest fill factor and efficiency [73]. Another study presents a new technique for preparing hexagonal-shaped mixed metal oxides (MMO) nanorods using Zn/Al-layered double hydroxide (LDH) as a precursor for dye-sensitized solar cell (DSSC) application. The molar ratio of Zn to Al significantly correlates with the nanorods' diameter and DSSC efficiency. The optical behavior and absorption enhancement of the MMO film are also demonstrated. The open-circuit voltage increases significantly with an increase in nanorod diameter, indicating that an increase in nanorod diameter is desirable due to enhanced surface area. The fabricated devices, ZA-8, exhibit the highest fill factor and efficiency. This work may provide an eco-friendly approach for future visible light photodetector applications [74].

**Fig. 4:**
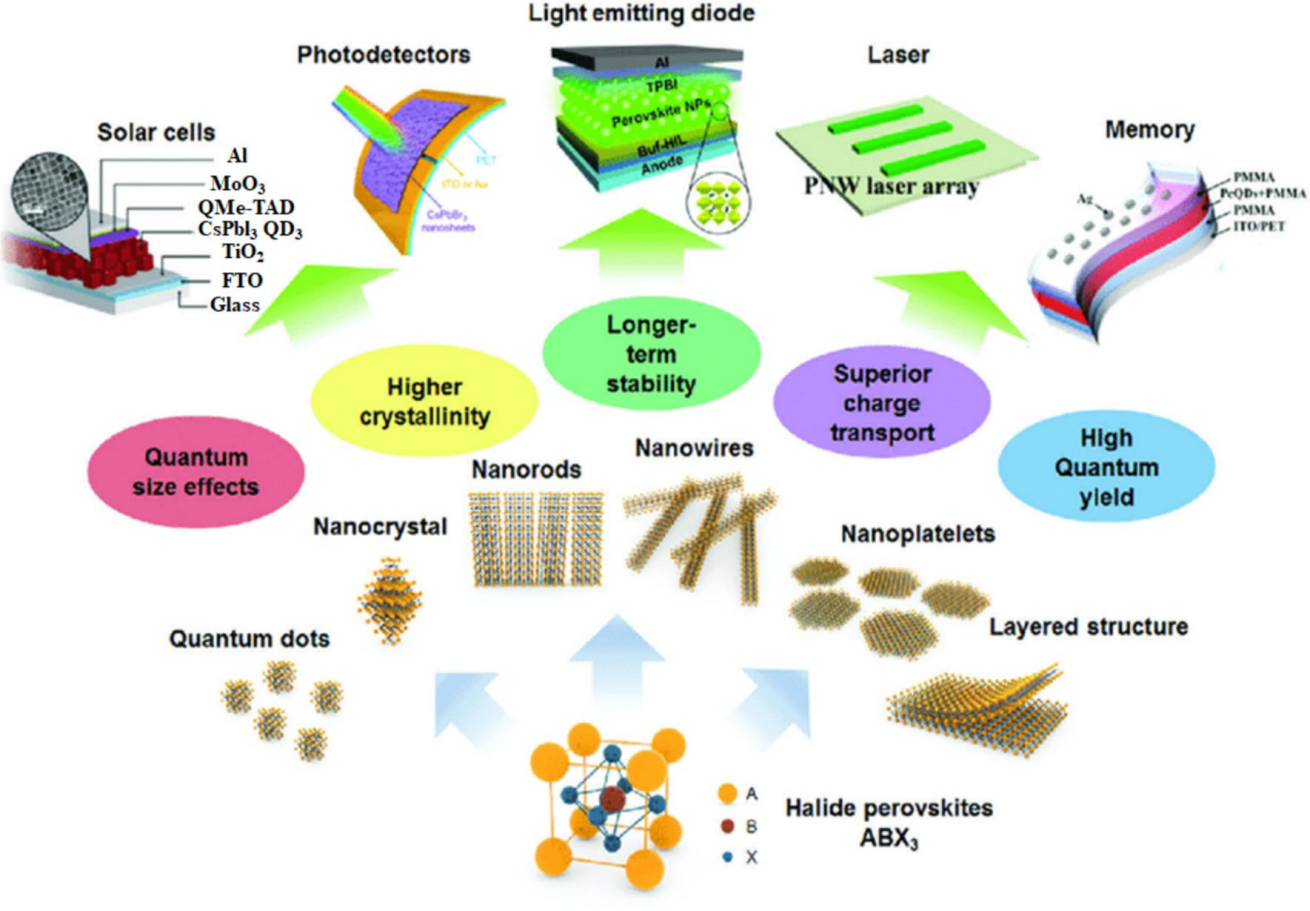
Schematic of various low-dimensional perovskite structures based on 2-D/3-D stacking and their applications. The different structure reveals unique properties as it is mentioned in the given figure. These perovskite materials serve in different forms of Solar Cells, Photodetectors, LED, Laser, and Memory reproduced with permission from Ref. [99]. © Copyright 2018, with the Royal Society of Chemistry publication

Currently, these semiconducting nanostructures in LEDs have become crucial because of their very particular optical properties. Optoelectronics has now focused on these nanostructure materials and the manufacturing processes of devices based on the improved nanostructures [75–77]. Nanostructures of various shapes can thus offer varying benefits to LEDs and are quickly gaining more attention as light-emissive substances that can help to increase the efficiency of the LEDs (Fig. 4). The substances could be developed to deal with the inefficiencies that are present in LEDs emitting UV and blue-violet spectrums [78–80].

The existence of low dimensional luminescent compounds such as calcium titanium oxide (CaTiO_3_) mineral perovskite has continued to show great potential in electronic devices. However, in optical aspects, their performance is not good enough when weighed against conventional organic LEDs. Thus, recent research studies have focused on exciton confinement to improve the highly effective perovskite LEDs [81–83]. Factors that affect the effectiveness of electroluminescent devices are a good photoluminescence quantum yield emission layer, better electron and hole injection layers, an electron and hole transport layer, and high light output coupling efficiency. Research and advancements in LEDs need the realization of emission layers from novel materials for better electron–hole recombination [84]. For better electron–hole recombination, the performance of an amorphous zinc-silica-oxide system that is structured around perovskite crystals is needed to strengthen the functioning of the diode [85, 86].

Shallow tunable electron affinity and large electron mobility allow amorphous zinc silicon oxide to capture and transport electrons. Structuring the amorphous zinc silicon oxide and the perovskite crystal enabled researchers to hold the excitons and inject the electrons into three-dimensional perovskite layers more efficiently [87, 88]. The energy level arrangements among the structures have offered a preferred material for this purpose. The blue, red, and green perovskite LEDs are referred to as PeLEDs [89–91]. The green diode was shown to be operating at the least voltage of about 2.9 V at 10,000 cd/m^2^ and worked more efficiently at 33 lm/W, and it was the brightest at 500,000 cd/m^2^. The results have shown a breakthrough in the manipulation of electron transport layer material, even though there are shortcomings, such as maintaining the stability of perovskite substances and removing the poisonous aspect of the lead in the crystal matrix [89, 92]. Despite these challenges, the results and outcomes offer novel opportunities to consolidate this methodology to reach more applications for perovskite LEDs in optoelectronics. The low dimensional perovskites are broadly categorized into 0D, 1D, and 2D perovskite [93, 94].

### Zero-dimensional perovskite

With the advancement in solvent-based synthesis methods, 0D inorganic perovskite such as Cs_4_PbBr_6_ has been synthesized in the form of nanocrystals or crystals, which contain the properties of organic molecules and inorganic semiconductors [95–98]. Cs_4_PbBr_6_ shows large exciton binding energy, intrinsic Pb^2+^ ion emission, and small polaron formation. For green photoluminescence, it shows high quantum yield and improved stability [98].

**Fig. 5:**
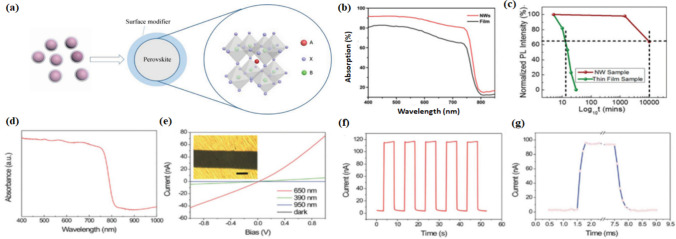
**a** Schematics of zero-dimensional perovskites reproduced with permission from Ref. [93] ©Copyright 2017, with the IOP Publishing. **b** Absorption spectra of perovskite films (in black colour) and NWs (in red colour) obtained from a mixed solution of Dimethylformamide (DMF) and Isopropyl alcohol (IPA) on a glass substrate. **c** Normalized Photoluminescence emission decay from Methylammonium Tin Iodide (MASnI_3_) thin film and NW samples against a logarithmic time scale reproduced with permission from Ref. [110] © Copyright 2018, with the Royal Society of Chemistry publication. **d** Ultraviolet–Visible (UV–Vis) spectra of Organic–Inorganic Perovskite Nanowire (OIP NW) films. **e** Response of photocurrent with different wavelengths. The inset is the optical image of a photodetector with a scale bar of 200 μm. **f** Current–time curve illuminated by 650 nm light at 2 V of bias. **g** High resolution scan to one cycle of current–time curves reproduced with permission from Ref. [110] © Copyright 2018, with the Royal Society of Chemistry publication

The 0-D perovskite (Fig. 5a), representative Cs_4_PbBr_6_, establishes a single crystal and powder form of high PLQY (approx. 48 percent). The pure phase of 0-D Cs_4_PbBr_6_ NCs has not been identified because the nucleation rate is in the uncontrollable form, which can be seen in traditional techniques [100, 101]. Hence using the reverse micro-emulsion technique for Cs_4_PbBr_6_, uniform distribution synthesis was reported with 26 nm size. By selecting a stoichiometric ratio, the impurity phase was eliminated. It leads to an 85 percent reaction yield for the 0-D Cs_4_PbBr_6_ rhombohedral phase [102, 103]. For white light-emitting applications, the 0-D inorganic perovskites’ properties were enhanced due to their self-trapped excitons exhibiting broadband emissions. The 0-D perovskite shows two types of hetero-structures with A_4_PBX_6_ and A_4_SnX_6_, and type-1 energy level alignment was reported by 0-D heterostructures. In these heterojunctions, energy was transferred from A_4_PbX_6_ perovskite to A_4_SnX_6_ perovskite. For white light-emitting applications, enhanced air stability has been reported [101, 104]. In bulk quantum-confined solids, to examine the lattice dynamics and behavior of charge carrier, the low dimensional perovskites are considered as leading materials in the application of optoelectronics. The 0-D inorganic perovskites Cs_4_PbX_6_ exhibit cubic or orthorhombic crystal structures. It contains Cs^+^ cations surrounded by the lead halide octahedral, and hence the 0-D shows single octahedron properties. The studies show that 0-D Cs_4_PbX_6_ 0-D perovskite exhibits low mobility and low electrical conductivity. The features of Cs_4_PbX_6_ perovskite, such as strong polaron localization and absorption of polaron band were revealed by transient absorption calculations and functional theory measurements. Due to octahedra's fast lattice relaxation and their weak interactions, short polaron lifetimes were observed [105, 106]. The 0-D Cs_4_PbBr_6_ perovskite shows green photoluminescence properties, which is highly efficient. Also, it has embedded intrinsic Br^−^ vacant states. From the embedded nanostructures, the Cs_4_PbBr_6_ crystals of emissive and non-emissive states were synthesized. Based on density functional theory measurements, the Raman modes of Cs_4_PbBr_6_ were assigned [107]. The presence of nanocrystals of Cs_4_PbBr_3_ in the emissive Cs_4_PbBr_6_ layer was revealed by Raman-PL [108, 109].

Due to their colour tunability capacity when solution processes, perovskites can address the bottlenecks of colour purity and efficiency. A resurgence in the study of 0D perovskite sparked with the solvent based synthesis. In 2016, inverse temperature crystallization (ITC), a method to grow different 3D perovskite single crystals was used to synthesize Cs_4_PbBr_6_ powder [111, 112]. Due to their insensitivity of emission towards temperature and form of material, i.e., powder or crystal, 0D perovskites have the advantage over other materials for reproducibility, which means 0D perovskites’ emission behaviour remains consistent under high temperatures and can be stored for a long time [113, 114]. The small and confined structure (discontinuous) of the 0D crystal can increase the effective mass for charge carriers, and in turn, strengthen electron and phonon coupling. This coupling facilitates the generation of small polaron. Due to the interaction of small polarons with lattice vibrations, energy levels are created and these energy levels form polaron bands also known as optical absorption bands. These absorption bands represent the presence of polarons and work as fingerprints of polarons. The energy levels, polaron bands, and fingerprints help to study the molecular behaviour of 0D perovskites. This molecular behaviour could be used to differentiate 0D perovskites from other low-dimensional perovskites. Thus, 0D perovskite materials are widely considered to be one of the most effective nanomaterials for photoelectric response devices because of their remarkable properties such as long photocarrier lifetime, fast charge transfer, simple processing technique and excellent stability (Fig. 5b and c). Cs_2_Snl_6_ nanocrystals’ broadband absorption and high absorption coefficient suggested that the present NWs were promising materials for high performance photodetectors, shown in Fig. 5d–g. 0D perovskites offer tunable emission, improved stability against moisture, oxygen, and light degradation, and can be synthesized using solution-based methods. They also exhibit high quantum efficiency, making them suitable for applications like LEDs and lasers. However, they face challenges in size uniformity, surface defects, limited stability, and synthesis complexity. Improving size uniformity is crucial for consistent device performance and efficiency. Surface defects can trap charge carriers, reducing quantum efficiency and non-radiative recombination processes. Addressing these challenges is crucial for commercialization and realizing the full potential of zero-dimensional perovskites in practical devices.

### One-dimensional Perovskite

Based on the solution process, the perovskite nanocrystals are synthesized, and they show emission color tunability, high luminescence quantum yield, and a sharp emission peak [115, 116]. One dimensional metal halide perovskite (MHPs) nanorods or nanowires do not have conventional cubic crystal structures, but it contains a corner-shaped metal octahedra. 1-D MHPs such as MAPbBr_3_ nanowires were prepared using a slip-coating process [117]. MAPbBr_3_ (Methyl Ammonium Lead Bromide) nanowire arrays were fabricated by Yang’s group and then converted them to MAPbl_3,_ (Methyl Ammonium Lead Iodide) without changing their morphologies, by anion exchange with MAI vapour. These nanowires exhibit properties such as high-quality factors and low thresholds [55, 118–121]. MASnI_3_ nanowires were fabricated using a porous alumina template (PAT), which was previously used to fabricate 3-D MASnI_3_ [122].

The hot-injection method is a common technique to grow 1-D halide perovskite, including CsPbX_3_ perovskites nanorods. In this method, cesium precursor is injected into the mixed solution of PbX_2_ (where X is either bromine or iodine) and octadecene (ODE) while using oleic acid (OA) and oleylamine (OAm) as assisting agent. During the hot-injection process, the solution is heated to a temperature to promote the formation of nuclei and to enhance the growth of 1-D perovskite structures. Wand and team prepared the Cs_2_SnI_6_ nanowires and nanorods by a process involving a reaction between tetravalent tin (IV) iodide and cesium oleate at 220 °C, using a mixture of OA and OAm [123]. The researchers were able to vary the morphology of the resulting Cs_2_SnI_6_ structures by adjusting the reaction time. Using oleic acid (OA) and oleylamine (OAm) as surfactants helps in stabilizing NWs and NRs by preventing them from agglomerating or growing too large. Cs_2_SnI_6_ is a potential material for perovskite LEDs and other optoelectronic devices, due to its good stability and high absorption coefficient and near-infrared regions of the spectrum [124, 125].

Organic–inorganic (OI) hybrid perovskites have gained a lot of attention, owing to their excellent optoelectronic properties, making them highly attractive for photovoltaics, LEDs, and other optoelectronic devices. One interesting phenomenon in these materials is the white light emission, arising from the self-trapped excitons in a lattice structure that are distorted [126]. A single-component hybrid perovskite which emits white light, such as CsH_14_N_2_PbCl_4_-H_2_O has different quantum-wire characteristics, generated by lead chloride octahedra, and has also been reported. When incorporated with pure enantiomeric organic cation, CsH_14_N_2_PbCl_4_-H_2_O becomes non-linear and gets circularly polarized. The interesting properties of CsH_14_N_2_PbCl_4_-H_2_O such as white light emissivity and chirality can help design the organic–inorganic perovskites to emit white light, which further can be used for many other applications [127, 128]. The synthesis of chiral and switchable ferroelectric 1-D halide perovskite has also been reported. The material exhibits these kinds of properties to facilitate the circular photogalanic effects and electric field and Rashba Dresselhaus splitting electric field and chiral enantiomer dependent [129, 130]. Through fast precipitation in an aqueous solution with an alkyl halide and no organic solvent, the 1-D organic–inorganic hybrid perovskite micro belt has been synthesized. The water stability and luminescent properties enhanced efficiently by protonated AD dyes integrated with perovskites system prevent water erosion too. The 1-D perovskites micro or nanostructure for strong micro devices optical communication applications extended due to the low loss co-efficient of optical waveguide performances, upconversion fluorescence, and polarized photoemission [131]. The synthesis methods, crystal structure, and properties of 1-D organic lead bromide perovskites, C_4_N_2_H_14_PbBr_4,_ have also been reported. Octahedral lead bromide chains are surrounded by organic cations to form the bulk assembly of core–shell quantum wires, enabling strong quantum confinement. The emission of bluish-white light with a good quantum efficiency (12% for bulk crystals and 12% for microscale crystals) is reported due to the self-trapped excited states formed [132]. 1D perovskites offer enhanced charge transport, improved stability, tunable optical and electronic properties, and easy synthesis. However, they face challenges in dimensional control, surface defects, limited charge carrier mobility, and device integration. The anisotropic structure of 1D perovskites facilitates efficient charge transport, making them ideal for applications in photodetectors, solar cells, and light-emitting devices. Surface defects can trap charge carriers, affecting device performance. Improving charge carrier mobility is crucial for high-performance optoelectronic devices. Lastly, device integration challenges must be addressed to transition 1D perovskite-based technologies from the laboratory to commercial applications.

### Two-dimensional Perovskite material-based LED

Quasi-2-D perovskites are not a type of perovskite material that has a layered structure, which leads to the formation of self-assembled quantum wells. These quantum wells can trap charge carriers, leading to efficient luminescence. In contrast to their 3-D perovskites, quasi-2-D perovskites have a reduced dimensionality in one direction, which can lead to improved stability and performance in optoelectronic applications. Quasi-2-D perovskite has shown promise in solar cells, LEDs, and photodetectors. The organic cations effect on quasi, the 2-D perovskite-based LED devices was reported [133].

**Fig. 6:**
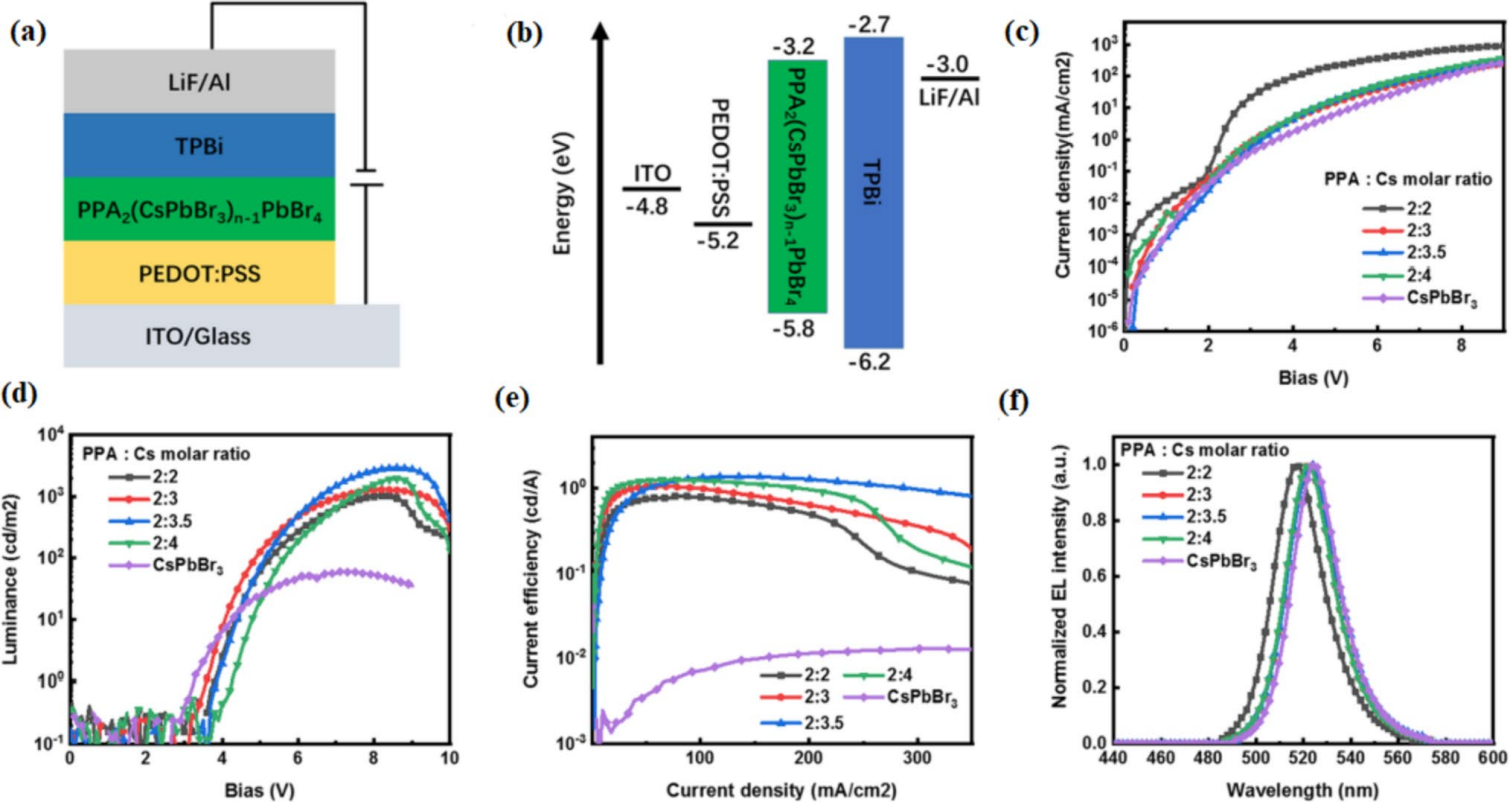
**a** Schematic representation of perovskites-based device. **b** The energy level of each layer used in the device. **c** Current density versus voltage curve, **d** luminescence versus voltage curve, **e** current efficiency versus voltage curve, **f** normalized EL (electroluminescence) intensity versus wavelength curve reproduced with permission from Ref. [135] © Copyright 2019, with the Springer publications

In quasi-2-D perovskite, usually consisting mixture of phases, strong exciton-phonon quenching occurs at room temperature, which shows low photoluminescence efficiency. To hinder the development of low-dimensional phase components, the proportion or molar ratio of cesium bromide (CsBr) and phenylpropyl ammonium bromide (PPABr) was adjusted. The brightness peak of 2921 cd per m^2^ and peak current efficiency of 1.38 cd A^−1^ were achieved using this optimized ratio. The peak current efficiency and brightness reported were way higher than the parameters obtained by conventional CsPbBr3 devices [134]. This exploration demonstrates another route for adjusting the stage structure in quasi-2-D perovskites to create exceptionally proficient perovskite-based light-emitting diodes (PeLEDs). This efficient method for achieving high-performance perovskite LEDs through such fabrication has the potential for mass development. The introduction of organic spacers such as (PPABr) has the potential to decrease the volume of the domain and heighten the surface coverage of perovskite film. The incorporation of moderated Cesium bromide in quasi-2-D perovskite shows better exciton lifetime and photoluminescence intensity. Thus, better PeLEDs that are structured around optimum Cs cations have been reported to show a higher brightness of 2921 cd per m^2^ and current efficiency of 1.38 cd A^−1^, as specified. This procedure may thus offer a breakthrough for making the PLQY of PeLEDs with quasi2-D perovskite film better (Fig. 6) [135].

Due to the advent of nanotechnology, the 2-D crystals of semiconductors including graphene, black phosphorous, and transition metal dichalcogenides offer a facile platform in LEDs due to their high carrier mobility, 2-D characteristics, band structure nature, and fabrication. The device structure can be constructed without lattice registration limits on any arbitrary stacking and for optoelectronics applications.

In recent years, two-dimensional (2D) metal halide perovskites have emerged as a potential material for high-performance light-emitting diodes (LEDs). When compared to other low-dimensional layered perovskites, 2D layered perovskite materials show excellent electrical and optical properties, which can be summarized as follows:

1. Dielectric and quantum confinement properties of the 2D perovskite layered structure help them to possess larger exciton binding energy (hundreds of meV), resulting in enhanced radiative recombination and thus higher PLQY in 2D layered perovskite-based devices.
2. Effective energy transfer from lower energy state quantum wall to higher energy state quantum wall due to the presence of cascaded or layered structure within 2D perovskite films. This swift and effective energy transfer helps in reducing the exciton quenching effect and enhancing the radiative combination.
3. When the main objective of the material is to provide swift energy transfer, environmental stability, and quantum confinement, then 2D perovskite with hydrophobic ligands is preferred over 3D perovskite with hydrophobic ligands.
4. The organic and inorganic subcomponents of 2D perovskite offer remarkable possibilities to finely adjust the optical and electrical properties, which makes them suitable for use in circular-polarized emission, detectors, and broadband emissions.

### Graphene-based Perovskite

**Fig. 7:**
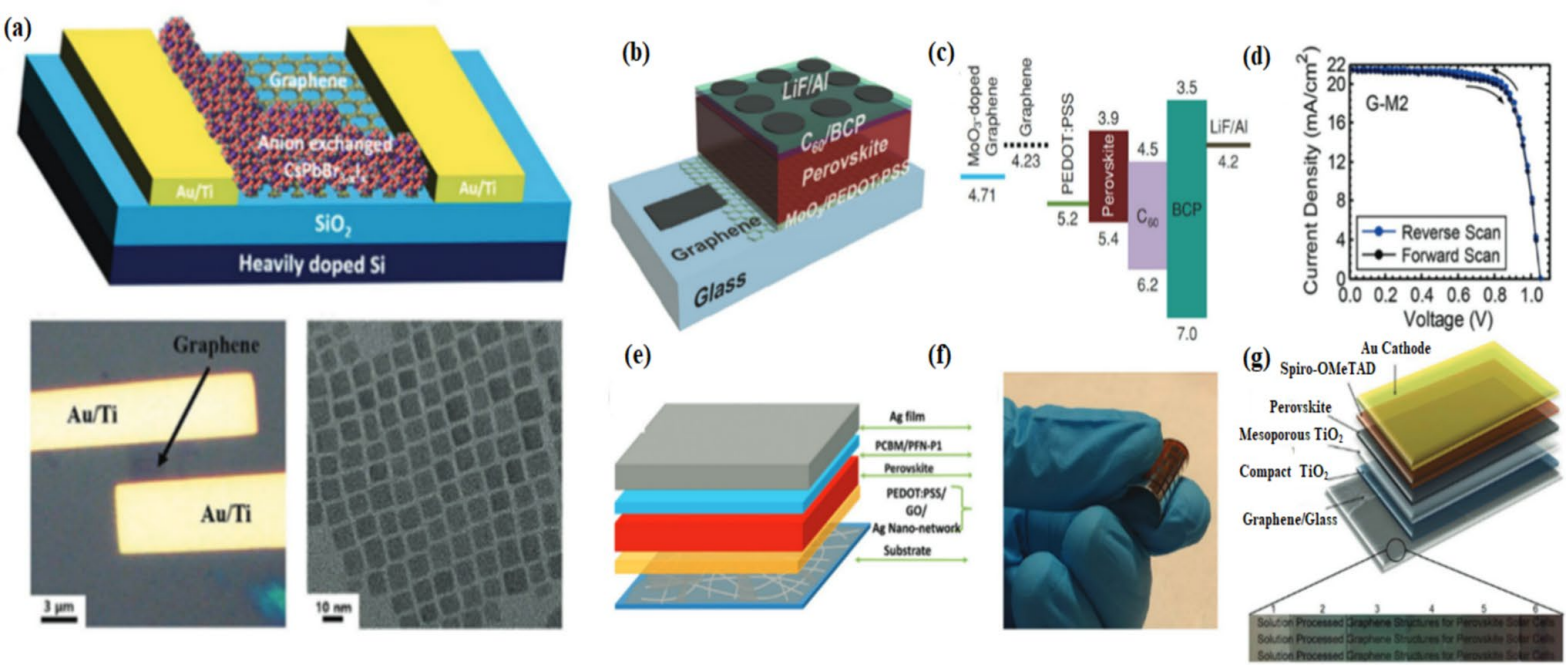
**a** Schematic of Graphene/MAPbl_3_-NW hybrid phototransistor. The bottom panel shows Graphene/CsPbBr_3_-xlx-NC hybrid phototransistors and graphene as TBEs for hybrid phototransistors and perovskite solar cells (HPSCs). **b** Schematic for chemical vapor deposition (CVD) based graphene TBE for HPSC. **c** Energy level diagram for graphene-based phototransistor. **d** Current density versus voltage curves for best performing device under AM 1.5G illumination at 100 mW cm^–2^. **a**–**c** Reproduced with permission. **f**, **g** Photograph of a flexible HPSC with Ag nanonetwork/rGO TBE on a PET substrate reproduced with permission from Ref. [140]. © Copyright 2017, with the Wiley–VCH publication

In 2-D hybrid organic lead halide perovskites, the emission of light from the R-NH_3_ 2PbX_4_ perovskite is tuned by using particular building blocks. These perovskites have a unique layered structure, consisting of PbX_2_ (inorganic lead halide) sheets sandwiched between an organic cation layer. The organic cations play a crucial role in determining the perovskite’s optoelectronic properties. Improved spatial resolution and excellent selectivity were reported when layered hybrid perovskites were tuned with non-covalent graphene. 2-D. Highly resolved perovskite phase spectra were obtained using a cryo-Raman spectrometer, where Raman mapping helped in tuning the perovskite and graphene constituents for resolved spatial spectra [136].The introduction of a graphene monolayer at the ZnO ETL (Zinc Oxide electron transfer layer) and perovskite absorber was reported to improve devices’ stability and photovoltaic performance, thereby enhancing their electrical efficiency by 19.81%. It also protects the electric decomposition on perovskite film and shows a greater advantage in device stability [137, 138]. 2D materials incorporated with graphene and perovskite quantum dots in a vertical phototransistor were reported to be more efficient light emitter and photodetector in a device in terms of ultra-sensitivity, ultrafast, and covering a broad band of light emission [139]. Another hybrid halide perovskite material *i.e*., Cesium lead halides CsPbX_3_ exhibits long carrier diffusion length and robust light absorptivity with enhanced stability. By depositing CsPbBr_3_-_x_l_x_ nanocrystals on exfoliated graphene over SiO_2_ /Si substrate, the graphene -CsPbBr_3_-_x_l_x_ nanocrystal hybrid phototransistor forms as shown in Fig. 7a. High carrier mobility and ultra-high performance is observed [140]. The performance of reduced graphene oxide (rGO) based transparent bottom electrodes (TBEs) is limited because of its low conductivity, but it is a bottleneck for the development of such high-quality solution-processed graphene TBEs, as shown in (Fig. 7b–g).

### Black phosphorous based perovskite

**Fig. 8:**
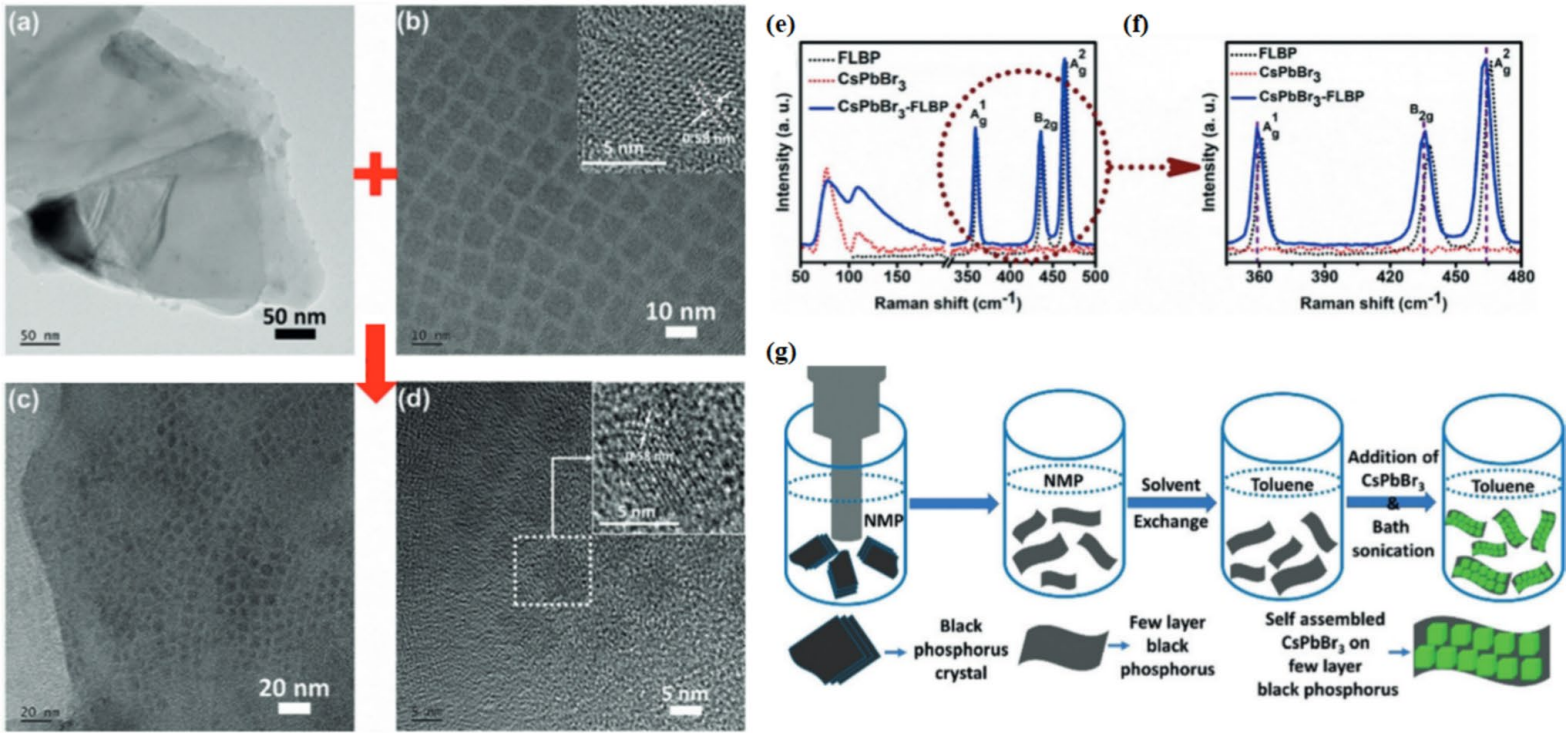
**a** High-resolution transmission electron microscopy (HRTEM) of FLBP sheets **b** High-resolution transmission electron microscopy (HRTEM) of CsPbBr_3_ QDs. **c** HRTEM images of the self-assembled CsPbBr_3_ QD **d** HRTEM images of FLBP nano-composite. **e** The Raman spectrum of FLBP (black line) shows signatures of A_1g_, B_2g_, and A_2g_ modes, the Raman spectrum of the CsPbBr_3_ QDs (red line) features bands at 77.3 cm^─1^ and 108.4 cm^─1^, and the Raman spectrum of the nano-composite (blue line) reflects the presence of QDs and FLBP sheets, with the same peak position as for the separate systems. **f** Enlarged Raman data reveals a definite shift, representing coupling strain effects originating from the integration of the CsPbBr_3_ QDs and FLBP. **g** Synthesis of self-assembled CsPbBr_3_ QDs on FLBP sheets reproduced with permission from Ref. [148]. © Copyright 2018, with the Wiley–VCH publication

Perovskite materials of different compositions and physiochemical properties show different efficiencies when fabricated as an LED device. 2-D materials as active layers have shown poor light absorption because of their thickness. Heterostructures of 2-D black phosphorous were introduced to overcome the light absorption capacity [141–143]. The black phosphorous single layer generally referred to as a Few layers of black phosphorous (FLBP) is considered as emerging material due to a tunable band gap and a unique anisotropic puckered mechanism, which was absent in graphene. The black phosphorous possesses high carrier mobility and it exhibits robustness towards optoelectronic applications. The low-dimensional materials incorporated into the heterostructure ensure the enhancement in the applications of optoelectronics [144]. Researchers observed a strong reduction (quenching) of QD fluorescence by incorporating ordered self-assembled CsPbBr3 quantum dots (QDs) on a few-layer black phosphorus (FLBP) surface. They used density function theory (DFT) calculations, time-resolved photoluminescence (TR-PL) studies, and photoconductivity measurement to analyze this quenching. TR-PL studies involve measuring the time-dependent behavior of the fluorescence emitted when QDs are excited by light. DFT calculations are a theoretical method that can be used to model the structure and its electronic properties. Photoconductivity measures the electrical conductivity of the material in response to light. The material configuration was achieved by utilizing self-assembly of CsPbBr_3_ QD over FLBP surfaces, as both the materials were prepared individually and combined in a particular ratio, by using a solvent suitable for both materials. Then CsPbBr_2_ QD was mixed with FLBP in a specific ratio sonicated at room temperature and finally incorporated into FLBP flat surface, shown in Fig. 8g. The experimental results are shown in (Fig. 8a) [145, 146]. The black phosphorous quantum dots BPQDs perovskite films were observed to exhibit a lower density of nonradiative defect, greater grain size and superior crystallinity, shown in Fig. 8a–d. The study establishes that during perovskite film formation, the BPQDs can be used as heterogeneous nucleation centers and lead to enhanced device performance, shown in Fig. 8e and f [147].

### h-BN based perovskite

**Fig. 9:**
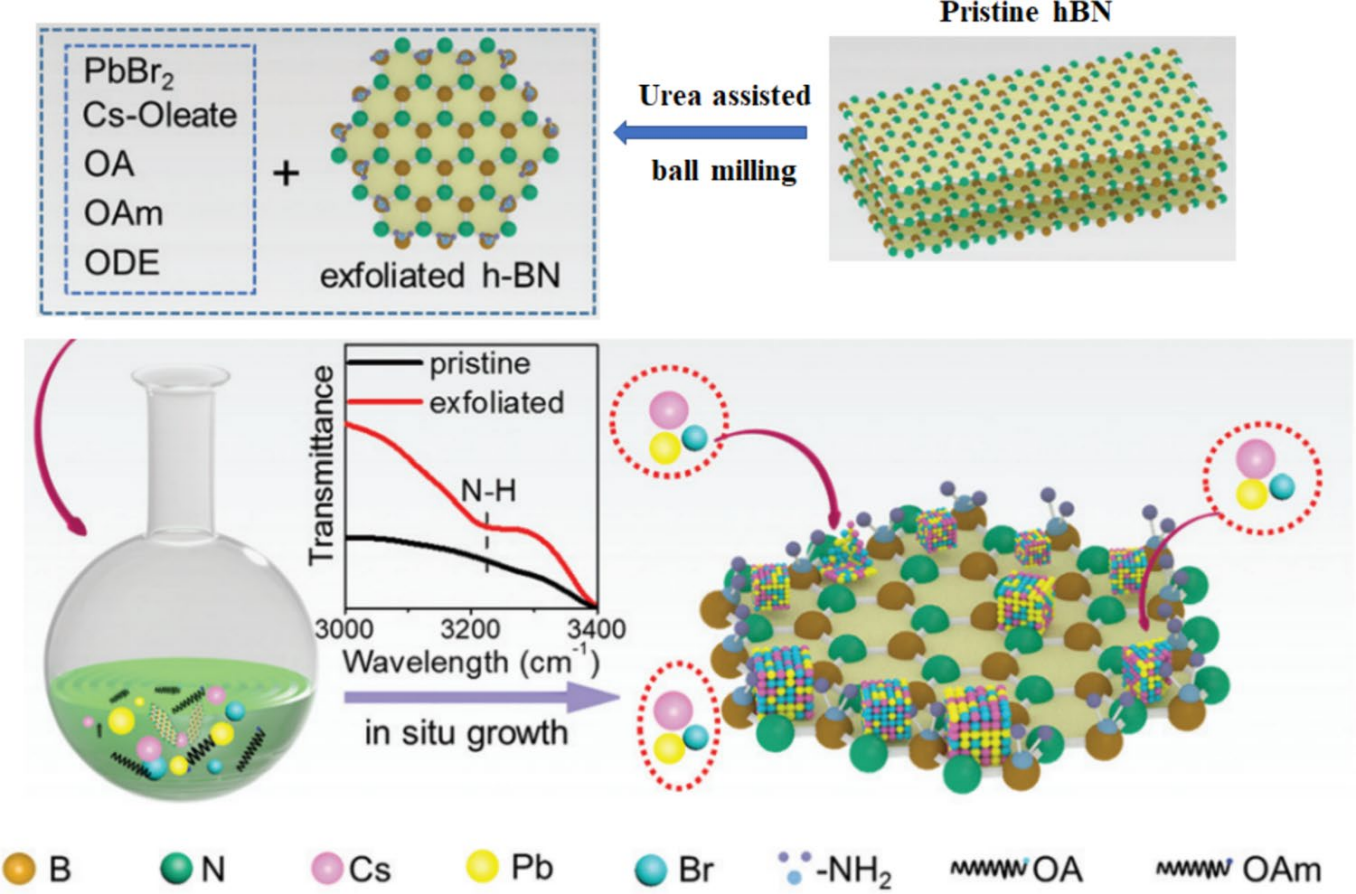
Illustration of direct growth and synthesis of nanosheet composites using CPB at BN-E composite powders. High thermally stable hexagonal boron nitride is exfoliated and then used as a substrate for perovskites to design extremely thermal stable LEDs reproduced with permission from Ref. [149]. © Copyright 2019, with the Royal Society of Chemistry publication

Sequential reactions in one single vessel (one-pot in-situ) strategy have been used for uniform perovskite nanocrystal growth directly onto an exfoliated 2D hexagonal-boron nitride nanosheets (h-BN NSs). This approach involves synthesizing the perovskite nanoclusters in the same reaction vessel as the h-BN NSs, which enables the growth of perovskite nanocrystals onto the surface of h-BN NSs. Thermal conductivity was observed to be more efficient in this nanohybrid structure and excess heat was dissipated timeously and efficiently. Most inorganic perovskite is based on lead halide materials which were reported to show the highest thermal stability, which leads to promising applications of perovskite material as seen in white LEDs and other optoelectronic devices [149]. Depositing Perovskite Nanocrystals on the surface of amino-modified h-BN white graphene (BNWG) yields high luminescence and ultra-stable phosphor material. The photoluminescence intensity attenuation was suppressed effectively and thermal stability was improved; hence, the white LED’s brightness and high stability were retained. The above-mentioned synthesis process shows simplicity and scalable production while maintaining its optical properties effectively. On the BNWG surface, the PNCs separate and produce multi-coloured light, highlighting the performance of PNCs-BNWG nanocomposites [150, 151]. Figure 9 shows the enhanced stability of luminescence of the composite material CPX h-BN designed through one pot situ growth.

### Transition metal dichalcogenides (TMDCs) based perovskites

**Fig. 10:**
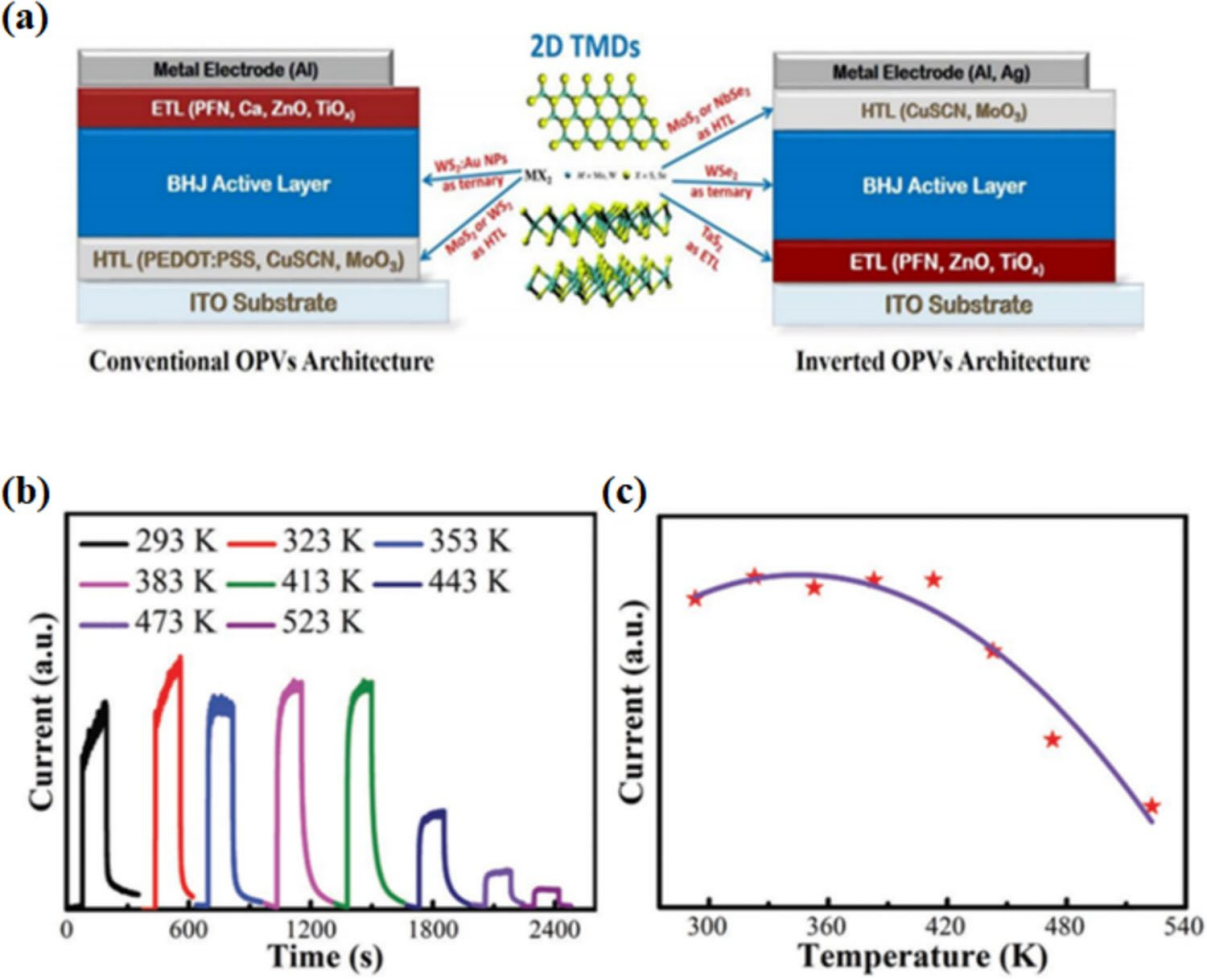
**a** Structure of 2-D TMDCs depicted in center and 5 blue arrows are the TMDCs various layers reproduced with permission from Refs. [155, 156]. © Copyright 2013, with the Springer Link and Wiley–VCH Publication. (**b** Current versus time response of hybrid perovskite (HP) at different temperatures. **c** Photocurrent response with temperature for the halide perovskite (HP)

TMDCs have been used as an emissive layer in hybrid organic–inorganic perovskite for the fabrication of LEDs. Higher efficiency and stability have been reported in LEDs using TMDCs based perovskite. TMDC materials like MoS_2_ and WS_2_ behave as charge transport carriers when sandwiched in a perovskite material. TMDC-based nanocomposites such as MoS_2_ nanosheets, embedded in perovskite material have shown better LED performance, owing to better transport properties and reduced recombination losses, compared to pristine perovskite-based LEDs [152, 153]. With 2-D semiconducting structure of transition metal dichalcogenides, the most significant nanomaterials considered were tungsten disulfide (WS_2_), tungsten diselenide (WSe_2_), molybdenum disulfide (MoS_2_) and molybdenum diselenide (MoSe_2_) exhibiting efficient optical, electrical and physical properties to be used in future optoelectronic and electronic applications. TMDs based optoelectronic devices exhibit efficient performances [107]. The main challenge exhibited by TMDs was their inefficiency in absorbing sufficient light due to their atomic thickness and preventing its integration into optoelectronic devices. This can be resolved by including an absorption layer with a high quantum efficiency (and high absorption rate), which can result in the TMDS having high-efficiency characteristics [154]. Figure 10 shows the architecture of Organic Photovoltaic Cells (OPV) with the presence of TMDs sandwiched between ITO substrate and metallic electrode, along with the photo response and dependence of annealing temperature over photocurrent.

## MoS_2_ based Perovskite

Remarkable properties like tunable band gap, better environmental stability, and the layered nature of MoS_2_ allow MoS_2_ to be implemented with perovskite materials for optoelectronic devices. Lead-free 2-D perovskite (p-type) heterostructures with few layers of MoS_2_ (n-type) photodetectors were reported, where the heterostructure performance is enhanced by adding few layers of graphene, resulting in superior performance over 3-D perovskite or 2-D photodetector material. From the MoS_2_ band edge, the heterojunction's spectral peak response shifts gradually. This structure can pave the way for 2-D perovskite optoelectronic applications [157, 158]. After annealing the HP at different temperatures, the temperature tolerance of the HPs is measured, as shown in Fig. 10b, c. The temperature tolerance shows that the PNC based 0D/2D HPs with high photo response exhibit good environmental stability. At zero gate voltages, the hybrid photodetector shows external quantum efficiency, high photo responsivity, and specific detectivity. It also exhibits operation durability and thermal stability, as the performances can be optimized by careful selection of material properties forming the heterojunction [156, 159, 160].

The 2D molybdenum disulphide (MoS_2_) as an active layer has been studied for application in optoelectronic devices. With 0-D CsPbBr_3,_ QD of 2-D monolayered MoS_2_ heterostructures have been prepared and optical features at the nanoscale are examined. The MoS_2_ used by field effect transistors and also CsPbBr_3_ QD MoS_2_ have been fabricated. In terms of charge transfer effect, photo responsivity, and electrical properties, the presence of TMDs greatly enhances field effect mobility and provides efficient energy transfer [161].

Two-dimensional metal halide perovskites have gained interest as a potential option for high-performance light-emitting diodes (LEDs). The 2D stacked perovskites are superior over other electroluminescent materials because of the layered structure's dielectric and quantum confinement, 2D perovskites typically have substantially larger exciton binding energies (hundreds of meV), which increases radiative recombination and, consequently large PLQY as shown in Table 2.

**Table 2:** Comparative study of PL emission and PLQY for CsPbX_3_ perovskite

Material	PL emission (nm)	PLQY	References
CsPb(Br/Cl)_3_	505	Up to 90%	[162]
CsPbX_3_ NCs (X = Cl, Br, I and mixed Cl/Br and Br/I systems)	419–520 and 522–670	Up to 86% and Up to72%	[163]
CsPbBr_3_	509–527	99.8%	[164]
CsPbX_3_(X = Cl, Br, I)	407–680	About 70%	[165]
CsPbX_3_(X = Cl, Br, I)-silicon resin composites	445–613	Up to 85%	[166]
CsPbI_3_	600	75%	[167]
CsPbX_3_ (X = Br, I) embedded phosphosilicate glasses	500–750	9.8–15.6%	[168]
CsPbBr3(In, Sb, and Bi)	499–513	57.2–91.2%	[169]
CsPb(Br/Cl)_3_ and CsPb(Br/I)_3_	410–700	20–80%	[170]
CsPbX_3_ mixed Cl/Br and Br/I systems	410–530	Up to 90%	[171]

Rapid and effective energy transfer from lower-n quantum wells to higher-n quantum wells (in sub-nanostructures) can be facilitated by the formation of cascaded energy structures within 2D perovskite films with mixed n (layer thickness). This can reduce the exciton quenching effect and enhance radiative recombination. The addition of hydrophobic organic ligands and the improved van der Waals contacts between the organic molecules of 2D materials lead to greatly increased ambient and heat stability. A considerably wider range of applications, including broadband emission, circularly polarized emission, and detection, are made possible by the exceptional chemical tunability of 2D perovskites, which includes both organic and inorganic subcomponents. 2D perovskites offer enhanced stability against moisture, heat, and light-induced degradation, making them suitable for long-term device applications. Their structural flexibility allows for precise control over bandgap energy, enabling tailored optical properties and absorption spectra. They also exhibit high charge carrier mobility, enabling efficient charge transport and extraction in optoelectronic devices. They can be synthesized using solution-based methods, making them compatible with low-cost fabrication techniques. However, they have several disadvantages, including anisotropic properties, interface engineering, environmental sensitivity, and limited structural diversity. Addressing these challenges is crucial for realizing the full potential of 2D perovskites in practical applications.

### Low dimension oriented organic–inorganic halide Perovskite

**Fig. 11:**
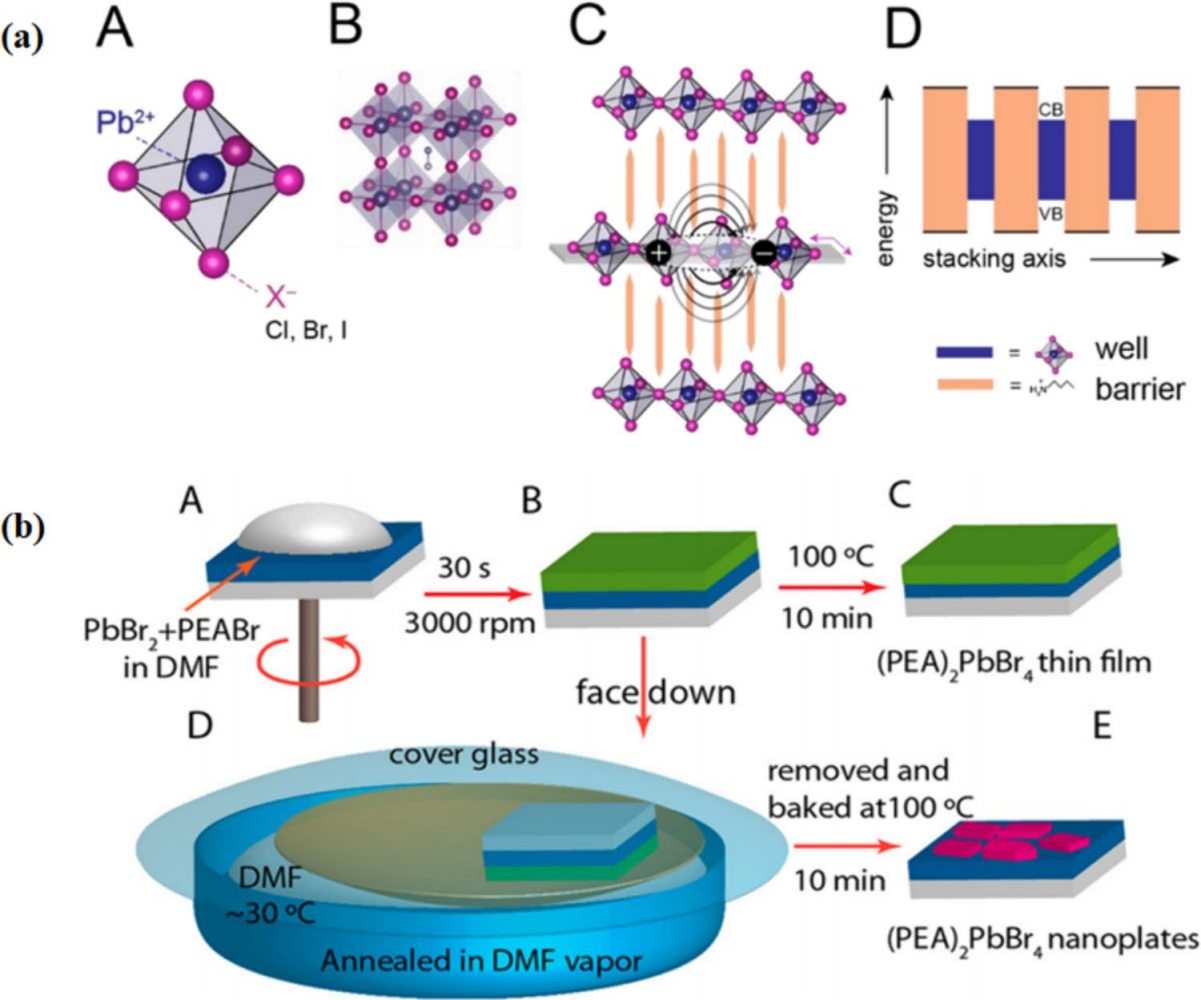
**a** Schematic diagram of lead halide perovskite shows dimensional tunability and natural quantum well systems. **A** perovskite structure basic unit is the (PbX6)_2_– octahedron, assembling into **B** the 3-D cubic perovskite structure, **C** with suitable stoichiometric preparation, 2-D layered perovskite structures self-assembles into **D** natural multiple quantum well structures reproduced with permission from Ref. [58]. © Copyright 2016, with the Elsevier publication. **b** Synthesis of polyethylammonium lead bromide (PEA)_2_PbBr_4_ thin films and nanoplates on” Poly(3,4-ethylenedioxythiphene)” (PEDOT): PSS-coated Indium tin oxide (ITO) substrates. **A**, **B** A dimethylformamide (DMF solution of 2: 1 PEA bromide (PEABr) and PbBr_2_ is spin coated onto a PEDOT: PSS (blue)-coated ITO substrate (gray). **C** or annealed in DMF vapor **D** and then baked at 100 °C for 10 min to prepare **E** micrometer-sized (PEA)_2_PbBr_4_ nanoplates (purple) reproduced with permission from Ref. [133]. © Copyright 2017, with the Wiley–VCH publication

There have been advances in the low dimensional oriented organic–inorganic halide perovskites that have progressed from their 2-D counterparts and also in their LEDs applications and usage as shown in Fig. 11a [172]. Recent studies have demonstrated electro-luminescent (EL) devices using 2D layered perovskite-based quantum well structures and phenyl–ethyl ammonium lead iodide in cryogenic temperatures [173]. Power conversion in LEDs in such experiments has been seen to be more efficient, 22% more than in perovskite-based solar cells with the utilization of 3-D perovskite with 2-D perovskite quantum confinement. In LEDs of methylammonium lead iodide MAPbI_3_‐based perovskite, reduced performance is linked with a decrease in exciton binding energy that is between 9 to 60 meV, which leads to free charge carriers and hence ends up in non-radiative recombination. Recent advances have been made to demonstrate that the use of phenyl methenamine lead iodide (C_6_H_5_CH_2_)_2_PbI_4_ at normal temperatures can be used as 2-D perovskites based green LEDs. Pirzado et al. developed an efficient antisolvent-assisted confined growth (AACG) method to fabricate high-quality formamidinium lead bromide (FAPbBr_3_) SCs at room temperature (RT). Efficient growth of high-quality OIHP-SCs at low temperatures paves the way toward the development of high-performance perovskite SC-based EL devices. These findings were able to show that the LED devices that had been layered with the material were able to exhibit an electroluminescence band at a wavelength of 0.526 microns and a narrow full-width half maximum (FWHM).

**Fig. 12:**
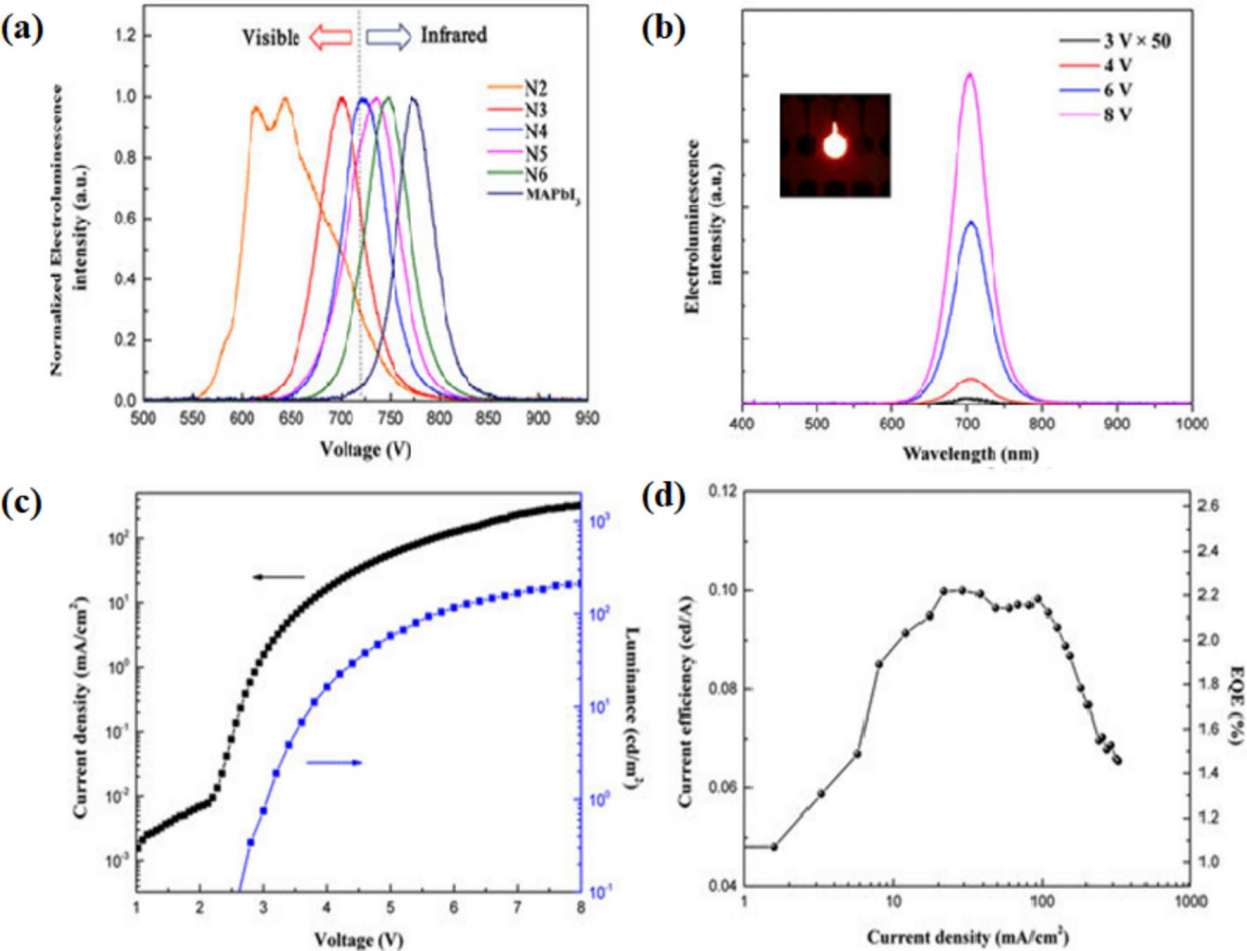
**a** Electroluminescence (EL) spectra of quantum well (QW) perovskites and MAPbI_3_. **b** Electroluminescence intensity of N_3_ LED under different voltages. Inset shows a N_3_ LED at 6 V. **c** Current density and luminescence versus voltage characteristics of N_3_ perovskite LED. **d** Current efficiency and External quantum efficiency (EQE) of N_3_ LED reproduced with permission from Ref. [58]. © Copyright 2016, with the Elsevier publication

Furthermore, quantum-confinement in 2-D perovskite structure (2- phenyl–ethyl ammonium lead bromide (PEA**⋅**PbBr_4_)) results in colour-pure violet LEDs (Fig. 11b) [174]. It was observed that the low-dimensional materials were able to come together in a proper restricted quantum well structure, by repeating a dielectric spacer and squeezing 3D PbBr_6_ in the device structures. LEDs with tunable bandgap and diverse photoluminescence regions can be achieved by using the 2 D perovskite structure of n = 1 (single layer) to n = 6 (six layers) through a spacer of butylammonium iodide (C_4_H_9_NH_3_I) [175]. The researchers associated with the wavelength at λ = 510 nm -the existence of an extreme retention band at roughly with a solid photon–exciton combination limited to the 2 D quantum wells. By PL spectra of films, the greater binding energy exciton described radiative recombination state. The mixture of halide hybrid perovskites has shown the formation of reversible traps and efficient device fabrication with broadband photo-detection [176, 177]. The best luminescence of 214 candelas per sq. meter was accomplished at 8 V, though the greatest external quantum efficiency (EQE) was 2.29%. By changing halide particles in the perovskite layer a transition from infrared to visible electroluminescence was observed (Fig. 12) [175].

## Three-dimensional Perovskite based LEDs

Despite the increased developments in the energy industry, perovskite-based LEDs have continued to attract the attention of scholars and other key players in the lighting industry [178, 179]. Perovskites such as organometal halides possess optoelectronic properties essential in semiconductors [180, 181]. Therefore, they significantly contribute to the chromaticity of LEDs [182]. Hybrid 3-D perovskites indicate good charge mobilities and high photoluminescence quantum efficiencies (PLQEs) [183, 184], thus making them significant in the electroluminescence (EL) aspect of LEDs. Three-dimensional (3D) perovskite structures exhibit high thermal stability thus enhancing the resistance qualities of LED lights [75, 185, 186]. Various technological advancements have been used to support the structural evolution of perovskites, consequently improving their optoelectronic properties. 3-D hybrid perovskites are semiconductors created through “solution-state film deposition [187]”. They consist of various optoelectronic characteristics, such as charge mobility, high carrier lifetime, direct band gaps, and high photoluminescence efficiency [188, 189]. These properties are vital in the advancements in LEDs, solar cell absorbers, and lasers [185, 187].

The compression of perovskites under different degrees of pressure enormously affects their structural transformations and as a result their electronic conductivity, structure, and color. Manipulation of these organic and inorganic hybrids has thus made it possible for lighting experts to discover more advanced developments of LED lights continuously. Metal halide perovskites exhibit high photovoltaic properties thus making them a central part of the development of LEDs and described as “crystalline materials originally developed out of scientific curiosity [190].” However, metal halide perovskites have, over the recent years gained popularity and become critical rivals of conventional photovoltaic technologies [190]. Organic–inorganic perovskites are comparable with photovoltaics [191] due to their good color purity and narrow emission linewidths of less than 20 nm and high PLQY (> 90%) in solution for nanocrystals) [181, 192].

A combination of several perovskites enables an increment in power conversion efficiency through significant adoption of updated deposition techniques and solid-state hole conductors [185, 193]. A comparative study of various PELEDs is given in Table 3. Therefore, perovskites continue to prove their promising characteristics relevant to LEDs. Enhanced stability and excellent power conversion efficiency of perovskites improve the durability of LED devices. Positively charged organic material (monovalent organic cation) based lead-halide perovskites show advanced solar cell performance and good charge transport properties [194, 195]. The use of monovalent ions also improves the optoelectronic properties of lead halide perovskites [196]. Thus, a combination of various perovskites enhances the general properties of LEDs developed, ultimately resulting in the creation of more reliable and long-lasting lighting products. Three-dimensional (3D) perovskites are promising optoelectronic materials for photovoltaics due to their high absorption coefficient, high charge carrier mobility, and tunability of bandgap. They can be synthesized using solution-based methods, allowing for scalable and cost-effective fabrication. However, they face stability issues due to exposure to moisture, oxygen, or light, resulting in reduced device performance and operational lifetime. Hysteresis in current–voltage characteristics can complicate device characterization and optimization. Lead toxicity concerns arise from the presence of lead in many 3D perovskites, raising concerns about environmental and health impacts. Material processing is crucial for achieving uniform and defect-free films and optimizing material processing parameters is essential for high-performance photovoltaic devices. Addressing these challenges is crucial for realizing the full potential of 3D perovskites in commercial applications.

**Table 3:** A comparative study of various LEDs

Structure	Max. LE (cd m^−2^)	Max. CE (cd A^−1^)	EQE	References
ITO/Buf-HIL(50 nm)/perovskite/TPBI (50 nm)/LiF(1 nm)/Al	2935	4.9	–	[197]
(ITO)/PEDOT:PSS/(PEA)2PbCl2Br2/TPBi/Ca/Al	70	–	–	[198]
ITO/PEDOT:PSS; ~ 40 nm)/7% Rb-incorporated FAPbBr3 film (~ 280 nm)/TPBi; 60 nm)/LiF (1 nm)/Al	66,353	–	–	[199]
(ITO)/PEDOT:PSS, 40 nm)/poly-TPD(20 nm)/EL(50 nm)/(TPBi)(40 nm)/LiF(1 nm)	1392	1.74	6.23%	[200]
All-inorganic CsPbBr3	12,650	13.43	4.33	[201]
ITO/PEDOT:PSS/perovskite/MoO3 (10 nm)/Ag (100 nm)	3780	6.1	5.70%	[202]
ITO/PEDOT: PSS (40 nm)/poly-TPD (30 nm)/CsPbBr3 QDs (20 nm)/TPBi (40 nm)/LiF/Al	15,185	13.33	6.27%	[203]
ITO/PEDOT:PSS/CsPbBr3/MABr perovskite/B3PYMPM/LiF/Al	3400	23	20.30%	[193]
ITO/PEDOT:PSS/Poly-TPD/CsPbBr3(DOAB)/TPBi/LiF/Al	990	31.7	9.70%	[204]
Al/n-ZnO NPs/CsPbBr3 QDs/p-NiO/ITO	6093	7.96	3.79%	[205]
ITO/PEDOT:PSS/Poly-TPD/IDA-treated CsPbI3 NCs/TPBi/LiF/Al	748	-	5.02	[206]
ITO/PEDOT:PSS/Perovskite A10CQW + PMMA/TPBi/LiF/Al	1288	30.4	7.37%	[207]
ITO/PEDOT:PSS (40 nm)/CsPbBr3 (200 nm)/TPBI (20 nm)/LiF (1 nm)/Al	16,436	32	10.50%	[208]
ITO/PEDOT:PSS/CsPbBr3 QDs/POT2T/Ca/Al	3880	9.22	2.64%	[209]
ITO/PEDOT:PSS/PEA2(FAPbBr3)n-1PbBr4/TPBI/LiF/Al	7829	52.51	12.12%	[210]

Buf-HIL ¼ buffer hole injection layer; PEDOT:PSS ¼ poly(3,4-ethylenedioxythiophene) polystyrenesulfonate; TPBI ¼ 2,20,200-(1,3,5-Benzinetriyl)- tris(1-phenyl-1-H-benzimidazole); B3PYMPM = C_37_H_26_N_6_; PEA = C_6_H_5_C_2_H_4_NH_3_; Poly TPD = Poly(bis-4-butylphenyl-N,N-bisphenyl)benzidine; PO-T2T = 1,3,5-triazine-2,4,6-triyl)tris(benzene-3,1- diyl)tris(diphenylphosphine oxide).

All inorganic PeLEDs rank among the recent advancements in LEDs that have been gaining significant attention [211]. Attention is attributed to the perovskite’s promising properties such as bright luminescence, outstanding operational stability, high efficiency, excellent color purity, bright luminescence and low cost of preparation [212, 213]. All-inorganic PeLEDs improve lighting device’s performance through various strengths such as balance and leakage, transport, and charge injection [214]. Due to increased technological advancement, people have continuously demanded the development of devices that are small in size to encourage portability. In this case, tuning band gaps using temperature changes facilitates controlling the dimensions of all-inorganic PeLEDs, thus enabling the creation of simple structures and flexibility and meeting the needs of the market. Semiconductors with low band gap are more suitable for solar light absorbers [215]. Such qualities allow for the expansion of the uses of perovskites, making them suitable materials for LEDs.

Exploration of all-inorganic PeLEDs unique properties facilitates device engineering [84, 216]. For instance, Lozano *et. al.* noted that PeLEDs were designed as “easy-to-prepare high-performance semiconductors” in 2008. They have impressive color purity and efficient color-tunability hence creating opportunities for new and easy-to-achieve lighting devices [217–219]. Incorporating these qualities with modern technologies, PeLEDs create avenues for exploration that indicate the possibility of more LED-associated inventions. Nano-structured and low-dimensional perovskites significantly contribute to the establishment of high-power applications [46]. Perovskites allow manipulation through changes in pressure and temperature, therefore enabling control of the elemental atomic structure [220]. These changes in electronic properties of materials impact not only the band gap but also band edge characteristics and consequently the nature and quality of light extracted and transmitted from them [221]. Developers of light products apply methods such as tailoring the chemical compositions of the materials used to enhance ways of processing, improve electrical output, and increase stability. Therefore, over the years perovskites have proved their critical roles in processing LED lights, thus indicating the need for continuous research and implementation in the industry. Such practices will ultimately contribute to enhanced, affordable, and environmental friendly developments that considerably meet the changing needs of the global population.

Light-emitting diodes are distinctively used in electrical and optical technology, which has been experiencing enormous growth in recent years. Various factors attributed to the increased growth of LED technology include high efficiency, durability, low power consumption, rugged construction, and reliability [222, 223]. LEDs are significantly used for various reasons such as traffic lights, large-area displays, aircraft lighting, automotive industry [224], and in agriculture, where LED is used as a source of light in greenhouses [225]. With the increased technological development and research, LED technology is bound to continue expanding its uses to more fields in the future.

## Prospective and challenges

The generation of green and red light through down-conversion of high-energy UV or blue light LED technology lowers the overall efficiency due to increased wastage of energy. With these inefficiencies, the light produced is cold white thus indicating low-quality [98, 226]. Low dimensional perovskites with considerable electron–hole binding energy are associated with energy losses which underscore the efficiency of LEDs. This situation often occurs with devices whose design implements free carrier flow such as solar cells and phosphors. Therefore, these challenges indicate the need for in-depth research to identify various ways of improving the performance and efficiency of LED technology [227, 228]. Different emission properties of LED materials affect the quality of light. Factors such as environmental parameters, concentration, temperature variations, and humidity affect the quality of light produced by LEDs [229]. Aluminum gallium nitride (AlGaN) optoelectronic devices are associated with challenges such as high device operation voltages and low internal quantum efficiencies (IQEs) [230, 231]. Consequently, these problems affect the technologies to achieve the desired LED characteristics.

The use of metal enhanced emission as a solution to high emissions in luminescent lighting is frequent in the light industry [232, 233]. The Ir (III) complex facilitates the development of new molecular imaging probes or enhances organic LED’s emission efficiency because of their improved optical properties. Using the Langmuir–Blodgett (LB) method, scientists demonstrate the nanometer-thick films to control emission wavelength. They act as oxygen sensors thus enabling the transfer of energy from the triplet excited state of the Ir(III) complex to either a semiconductor or oxygen molecule in a triplet ground state [232, 234]. Its applications thus allow the users to control the needed emission wavelength spectral range. Further challenges are experienced in controlling the ratio of different colors in luminescent lights. However, the adoption of technologies such as photonic crystals, plasmonic, and quantum dots enables us to overcome this challenge [232, 235, 236]. For instance, photonic crystals and nanostructures, such as multilayers, gratings, and thin films have structural colors based on their respective size of the nanostructure, the angle of incidence, and refractive index. Understanding each material’s properties enables efficient control of colors based on the desired outcomes [237–239].

Additionally, other properties that impact the quality of light in LEDs include a mismatch between substrates such as SiC and AlN, which increases dislocation densities [240, 241]. The use of these substrates also reduces the reliability and efficiency of the LED devices developed [242, 243]. The low internal quantum efficiency (IQE) of LEDs limits the technological outcomes of advancements in LEDs [244]. Variables such as high pressure lower the quality of AlN thin film growth thus impacting the outcome of the processes [2, 245, 246]. These variations and issues in the production of LEDs are time-consuming and require the incorporation of multiple technologies, knowledge, and skills which are expensive and hard to acquire. Substantial manufacturing and consumption costs of LEDs undermine the exploration of new materials thus ultimately affecting the rate of innovation and invention in the lighting industry. High costs rank among the elements that undermine maximum the benefits of LED technology [247–249]. The acquisition of relevant modern technologies in the energy industry requires a lot of capital thus hindering innovation [250–252]. However, consistent diversification in the lighting industry through the adoption of solid-state lighting is expected to enable lowering the costs, primarily through reduced energy consumption [253, 254]. The future of LED technology is uncertain thus hindering people’s capability to embrace it openly [255, 256]. LED lighting is eco-designed to facilitate the reduction of the use of and dependency on metals thus creating an uncertain situation [256, 257]. The ultimate goal of this method of light production and consumption is to reduce environmental hazards that pose threats to the well-being of the people [258, 259]. Although the energy industry has been developing relevant strategies to incorporate the requirements of reducing emissions and lowering the use of metals in lighting, sustainable measures are yet to be identified and implemented [255, 260].

Perovskite-based LEDs have become a promising material for solid‐state lights and high‐definition displays due to their high PLQY, tunable emission wavelength, and narrow emission linewidths [261, 262]. In recent years, the advancements in green and red emissive perovskite-based light emitting diodes have rapidly increased, also the corresponding external quantum efficiencies have exceeded by 20%. The defects and traps and narrow band gap of the crystal structure negatively affect the electron–hole combination and charge transport, and therefore the blue light emissive perovskite has low efficiency [263].

The 2D materials of perovskites are considered the better material when compared to 0D and 1D perovskite materials. The 1D perovskite material application is limited to a selected few devices due to its cumbersome morphological characteristics and poor surface coverage which adversely affects the performance of the devices [236]. Similarly, the 0D perovskite material of random orientation and long chain excess in organic ligands on quantum dots surface leads to charge carrier mobility reduction of film of quantum dots. To enhance the performance the organic ligands, have to be forcefully removed. Due to several challenges faced in 0D and 1D materials of perovskite, 2D materials are considered suitable for LEDs for optoelectronic applications. Also, when compared with 3D perovskites the 2-D perovskites show increased stability. The following are the points at a glance regarding perovskite LEDs-

1. When compared with 0-D halide perovskites, the 1-D halide perovskite materials show improved thermostability, photostability, and high melting point. Unique features of one-dimensional halide perovskites nanocrystals exhibit higher promise for practical applications in optoelectronic devices such as lasers, LEDs, solar cells, and photodetectors. Bottlenecks like environmental stability, toxicity, and synthesis, should be addressed before 1D halide perovskites are considered for practical application.
2. In addition, LED application facilitated by 2-D perovskites has increased radiative recombination efficiencies. The challenges like the poor quality of thin film used in the fabrication of devices can be improved by using the technology to increase the surface area for 2D perovskite and minimize the defects and traps, such as surface passivation technique and film-assembly technique.
3. Multidimensional (2D, 1D, and 0D) perovskites, superlattices, heterojunctions, and doped structures can be developed using molecular beam epitaxy (MBE), which is used to synthesize the halide perovskite. Using high-pressure techniques to synthesize perovskites can also pave paths for improvements in LEDs and other devices as well.
4. Maintaining high device performance along with good environmental stability still remains a challenge in 2D perovskite devices. More efforts and research should be put in this direction to optimize the device's performance.
5. Computational models that can predict device performances based on the designs of the device, transport layers, active materials, and synthesis methods will come in handy in research and development in optoelectronic devices. The models will save time and resources and will give a better insight into the devices.

## Conclusion

LEDs are currently considered the most promising technologies in the lighting industry. Their efficiency and longevity are unparalleled in the world of illumination. Through the proper and widespread application of LEDs, individuals, as well as organizations, will benefit. Access to improved lighting devices that can be customized to play multiple functions. Although LEDs are one of the best modes of light generation on an industrial scale still there are shortcomings in their overall design. One of the notable problems regarding LEDs is the heat generation and dissipation of heat. The durability and longevity of LEDs decrease if the generated heat is not properly dissipated. The performance and energy efficiency of the LEDs can be further optimized by incorporating 2-D perovskite materials in LED research.

This work gives an overview of the current research regarding the improvement of LEDs by integrating 2-D perovskite materials. The authors believe that the review will provide a significant and compound overview of the potential of two-dimensional perovskites for LEDs, that can extend the material frontiers in photonic technologies. Graphene, Black phosphorous, and hBN-based perovskites have been discussed. Several current works have been incorporated into this study which are related to 0-D, 1-D, and 2-D perovskite materials. The prospects and challenges of perovskites and 2D- perovskites are discussed in detail. The authors hope this work shall be beneficial to researchers who are starting their work in this field and also to seasoned scientists who desire a brisk and updated survey of this field.
